# Coordination and Hydrogen Bond Chemistry in Tungsten Oxide@Polyaniline Composite toward High‐Capacity Aqueous Ammonium Storage

**DOI:** 10.1002/smll.202405592

**Published:** 2024-08-18

**Authors:** Shuai Mao, Xu Han, Zi‐Hang Huang, Hui Li, Tianyi Ma

**Affiliations:** ^1^ Institute of Clean Energy Chemistry Key Laboratory for Green Synthesis and Preparative Chemistry of Advanced Materials of Liaoning Province College of Chemistry Liaoning University Shenyang 110036 China; ^2^ Engineering Laboratory of Advanced Energy Materials Ningbo Institute of Materials Technology and Engineering Chinese Academy of Sciences Ningbo 315201 China; ^3^ Centre for Atomaterials and Nanomanufacturing (CAN), School of Science RMIT University Melbourne VIC 3000 Australia

**Keywords:** aqueous ammonium storage, charge storage mechanism, coordination, hydrogen bond chemistry, tungsten oxide

## Abstract

Aqueous ammonium ion batteries (AAIBs) have garnered significant attention due to their unique energy storage mechanism. However, their progress is hindered by the relatively low capacities of NH_4_^+^ host materials. Herein, the study proposes an electrodeposited tungsten oxide@polyaniline (WO_x_@PANI) composite electrode as a NH_4_^+^ host, which achieves an ultrahigh capacity of 280.3 mAh g^−1^ at 1 A g^−1^, surpassing the vast majority of previously reported NH_4_^+^ host materials. The synergistic interaction of coordination chemistry and hydrogen bond chemistry between the WO_x_ and PANI enhances the charge storage capacity. Experimental results indicate that the strong interfacial coordination bonding (N: →W^6+^) effectively modulates the chemical environment of W atoms, enhances the protonation level of PANI, and thus consequently the conductivity and stability of the composites. Spectroscopy analysis further reveals a unique NH_4_^+^/H^+^ co‐insertion mechanism, in which the interfacial hydrogen bond network (N‐H···O) accelerates proton involvement in the energy storage process and activates the Grotthuss hopping conduction of H^+^ between the hydrated tungsten oxide layers. This work opens a new avenue to achieving high‐capacity NH_4_^+^ storage through interfacial chemistry interactions, overcoming the capacity limitations of NH_4_^+^ host materials for aqueous energy storage.

## Graphical abstract

A tungsten oxide@polyaniline composite material modified by interfacial chemistry is designed for aqueous NH_4_^+^ ion storage for the first time. The ultrahigh capacity of 280.3 mAh g^−1^ at a current density of 1 A g^−1^ exhibited by the electrode is attributed to the effects of interfacial chemistry. The coordination chemical environment between the interfaces leads to enhanced Faraday redox behavior, while the presence of hydrogen bond chemistry induces a unique NH_4_^+^/H^+^ co‐insertion mechanism.

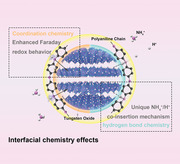

## 1 Introduction

Aqueous ammonium ion batteries (AAIBs) with nonmetallic ions as charge carriers have garnered significant attention in the field of energy storage, owing to their unique hydrogen bond chemistry between NH_4_^+^ and host materials, faster ion diffusion ability, lower cost, and higher safety compared to metal‐ion charge carriers (Li^+^, Na^+^, K^+^, Zn^2+^, etc.) in aqueous electrolytes.^[^ ^1^, ^2^, ^3^, ^4^, ^5^, ^6^, ^7^, ^8^ ^]^ To date, substantial efforts have been dedicated to developing NH_4_^+^ storage materials. Cui and co‐workers were the first to report the intercalation behavior of NH_4_^+^ ions in Prussian blue analogs (PBAs).^[^ ^9^ ^]^ Inspired by this groundbreaking work, Ji and co‐workers assembled the first “rocking‐chair” NH_4_^+^ battery and proposed a monkey‐swing model for energy storage to further elucidate the rich hydrogen‐bonding chemistry between NH_4_^+^ and bilayer V_2_O_5_. Unlike the energy storage mechanism of metal charge carriers, this unique hydrogen bonding interaction results in superior rate performance.^[^ ^10^ ^]^ Recently, Mai et al. further analyzed the deprotonation and hydrolysis reaction processes of NH_4_^+^ occurring at electrode–electrolyte interface and demonstrated reversible NH_4_^+^/H_3_O^+^ co‐insertion/extraction in VO_2_(B). This interface chemistry effect stabilizes the crystal structure of host material and enables fast rate performance in mild electrolytes.^[^ ^11^ ^]^ Although various materials have been reported for NH_4_^+^ energy storage to date, including Prussian blue analog (NH_4_)_1.47_Ni[Fe (CN)_6_]_0.88_, MXene, V_2_O_5_, MoO_3_, MnO_x_, and covalent organic frameworks (COFs), the issues of suboptimal specific capacity persist.^[^ ^5^, ^10^, ^12^, ^13^, ^14^, ^15^, ^16^, ^17^, ^18^, ^19^, ^20^ ^]^ Therefore, the development of innovative high‐capacity material or NH_4_^+^ storage chemistry is crucial for advancing the progress of AAIBs.

Composite electrodes characterized by interfacial chemistry have been extensively reported in various energy storage devices, where the strong interactions at the interfaces effectively modulate their electrochemical behavior. A coordinate bond is a special type of covalent bond in where the shared pair electrons are provided by the ligand and occupies the empty orbital of the central metal atom. This induces unique charge transfer and electronic effects between the two bonded atoms.^[^ ^21^ ^]^ Li et al. have consistently reported the excellent capacitance performance of NiFe LDH@PBAs and LCCH‐S(Se)@PBAs core‐shell hybrid electrodes. They demonstrated that the formation of strong interfacial coordination bonds between the two phases, promotes electron transport and enhances conductivity.^[^ ^22^, ^23^ ^]^ Unlike the strong covalent bonds, hydrogen bonds are weak interactions between highly electronegative atoms with lone pair electrons and hydrogen nuclei, characterized by saturation and directionality. The hydrogen bonding network within the electrode material significantly influences charge carrier transport, activates Grotthuss conduction between H_2_O molecules and drives rapid proton storage.^[^ ^24^ ^]^ Using vanadium oxide and manganese oxide as examples, Mai et al. proposed a general strategy for constructing hydrogen bonding networks within electrode materials to promote efficient charge carriers diffusion and enhance proton storage in aqueous zinc ion batteries.^[^ ^25^ ^]^ The aforementioned works suggest that coupling and rationally modifying multiple interfacial chemistry interactions within composite materials could be a promising strategy for achieving excellent NH_4_^+^ storage performance.

Given that the repeating unit structure of polyaniline is rich in amino functional groups which can provide both lone pair electrons as ligands and protons as hydrogen bond donors, modifying transition metal oxides with high‐valence central metal atoms using polyaniline may pave the way for diverse interfacial chemistry. In this study, a tungsten oxide@polyaniline composite electrode (WO_x_@PANI) was designed using electrochemical methods and applied for aqueous NH_4_^+^ storage for the first time. This electrode achieved an ultrahigh capacity of 280.3 mAh g^−1^ at 1 A g^−1^, surpassing most published materials for aqueous NH_4_^+^ storage. Moreover, the composite electrode demonstrates good cycling stability, retaining 83.3% of its capacity after 1000 cycles. These excellent electrochemical properties arise from the interplay of coordination chemistry and hydrogen bonding interactions. Experimental results indicate that the strong coordination interactions and hydrogen bonding network at the interface of the two phases can effectively enhance the protonation degree of PANI, activate Grotthuss conduction, and promote the NH_4_^+^/H^+^ co‐insertion process. When assembling the full AAIB using a CuFe PBA cathode, the device achieves an impressive energy density of 53.3 Wh kg^−1^ at 336.9 W kg^−1^, surpassing other previously reported AAIBs.

## 2 Results and Discussion

### 2.1 Construction and Characterization of Multiple Interfacial Chemistry

**Figure 1:**
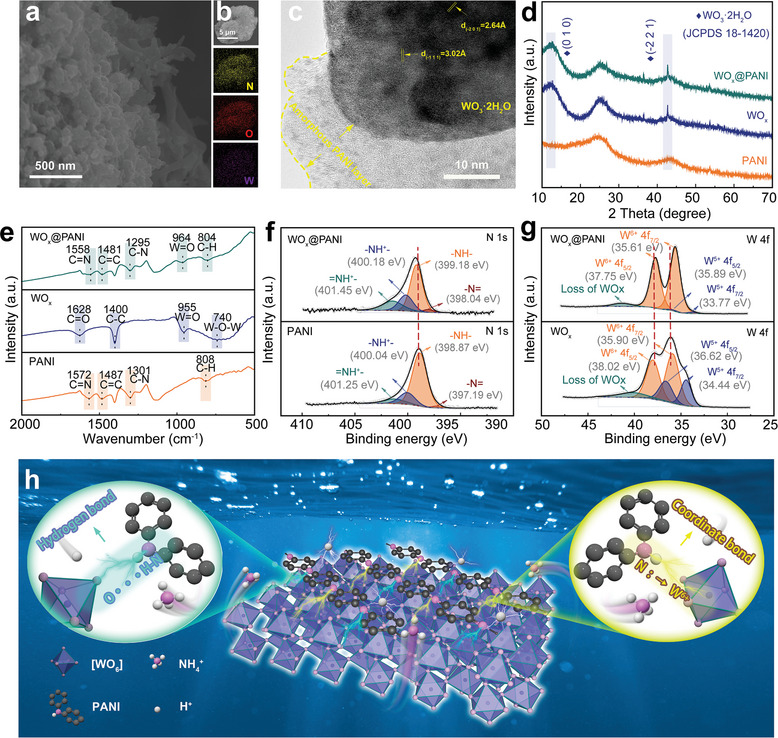
a) SEM image. b) Element mappings, and c) HRTEM image of WO_x_@PANI. d) XRD patterns and e) FT‐IR spectra of WO_x_@PANI, WO_x_, and PANI. f) XPS N 1s spectra of WO_x_@PANI and PANI. g) XPS W 4f spectra of WO_x_ and WO_x_@PANI. h) Schematic illustration of the coupling of multiple interfacial interactions of WO_x_@PANI.

After functionalizing the carbon cloth to enhance wettability and conductivity for electrodeposition, the tungsten oxide (WO_x_) is uniformly electrodeposited on the substrate using a constant potential method.^[^ ^14^, ^26^, ^27^ ^]^ Scanning electron microscopy (SEM) images revealed that WO_x_ grew uniformly on the carbon cloth fibers, displaying a cauliflower‐like morphology (Figure S1, Supporting Information). This morphologic feature enhances the electrode's surface area in contact with the (NH_4_)_2_SO_4_ electrolyte and accelerates the diffusion kinetics of NH_4_^+^. Subsequently, the aniline monomer rapidly forms a PANI film on the surface of WO_x_ (denoted as WO_x_@PANI) when subjected to a high potential. SEM images of WO_x_@PANI reveal that the original morphology of WO_x_ is largely preserved. Additionally, coral‐like nanorod structures appeared in some areas, which can be ascribed to the particular morphology resulting from the polymerization of PANI at high potentials (**Figure** **1**a). We performed the electropolymerization of PANI on a carbon cloth substrate and observed the same phenomenon (Figure S2, Supporting Information). This unique structure allows the PANI chains to intertwine and connect with the WO_x_ particles, filling the gaps between them. This further reduces the distance to the electrolyte and improves ionic transfer efficiency during electrochemical reactions.^[^ ^28^ ^]^ The elemental mapping images (Figure 1b) reveal a uniform distribution of N, W, and O elements. The high‐resolution transmission electron microscopy (HRTEM) image shows a clear delineation between the amorphous PANI layer and WO_x_ (Figure 1c). Moreover, the lattice stripe spacing observed in Figure 1c, corresponding to the (−111) and (−201) crystallographic facets of WO_3_·2H_2_O, is highlighted and aligns with the XRD data presented below.

To reveal the interfacial interactions between WO_x_ and PANI, a series of characterizations were conducted on WO_x_, PANI, and the WO_x_@PANI composite sample. Figure 1d compares their X‐ray diffraction (XRD) patterns. For PANI, no diffraction peaks other than those of the carbon cloth substrate were observed, confirming its amorphous nature. In contrast, the diffraction peaks at 12.7° and 43.0° can be recognized in both WO_x_ and WO_x_@PANI, corresponding to the (010) and (−221) planes of WO_3_·2H_2_O, respectively. The weak and broad diffraction peaks of WO_3_·2H_2_O indicate the low crystallinity of materials prepared by electrodeposition. The Fourier transform‐infrared (FT‐IR) spectrum of the electrodes are presented in Figure 1e. The FT‐IR spectrum of WO_x_@PANI exhibits characteristic absorption bands at 804 cm^−1^ (C‐H deformation vibrations out of the aromatic ring), 1295 cm^−1^ (C‐N stretching vibration in aromatic amines), 1481 cm^−1^ (C═C benzene ring stretching vibration), 1558 cm^−1^ (C═N quinone ring vibration), and 964 cm^−1^ (W═O), indicating the successful combination of WO_x_ and PANI.^[^ ^29^, ^30^ ^]^ Due to appropriate oxygen functional groups were introduced to the surface of carbon fiber to enhance its wettability, the peaks at 1400 and 1628 cm^−1^ are corresponding with the stretching vibrations of C‐C and C═O from functionalized carbon cloth, respectively.^[^ ^14^ ^]^ Notably, several peaks of the composite electrode red‐shifted to lower wavenumbers compared to C‐H (808 cm^−1^), C‐N (1301 cm^−1^), and C═N (1572 cm^−1^) stretching modes of the pure PANI electrode. Additionally, the characteristic peaks of the W═O (955 cm^−1^) band in the pure WO_x_ electrode blue‐shifted after electrochemical deposition. The peak shifts in the FT‐IR spectra indicate the formation of H‐bonding interactions (N‐H···O) between adjacent metal oxides and organic polymer modules.^[^ ^31^, ^32^, ^33^, ^34^ ^]^ This is more clearly demonstrated in the FT‐IR spectra of WO_x_@PANI (Figure S3, Supporting Information), where a characteristic signal is observed at a wavelength of 3014 cm^−1^, corresponding to the H‐bond stretching method (N‐H···O).^[^ ^31^ ^]^

**Figure 2:**
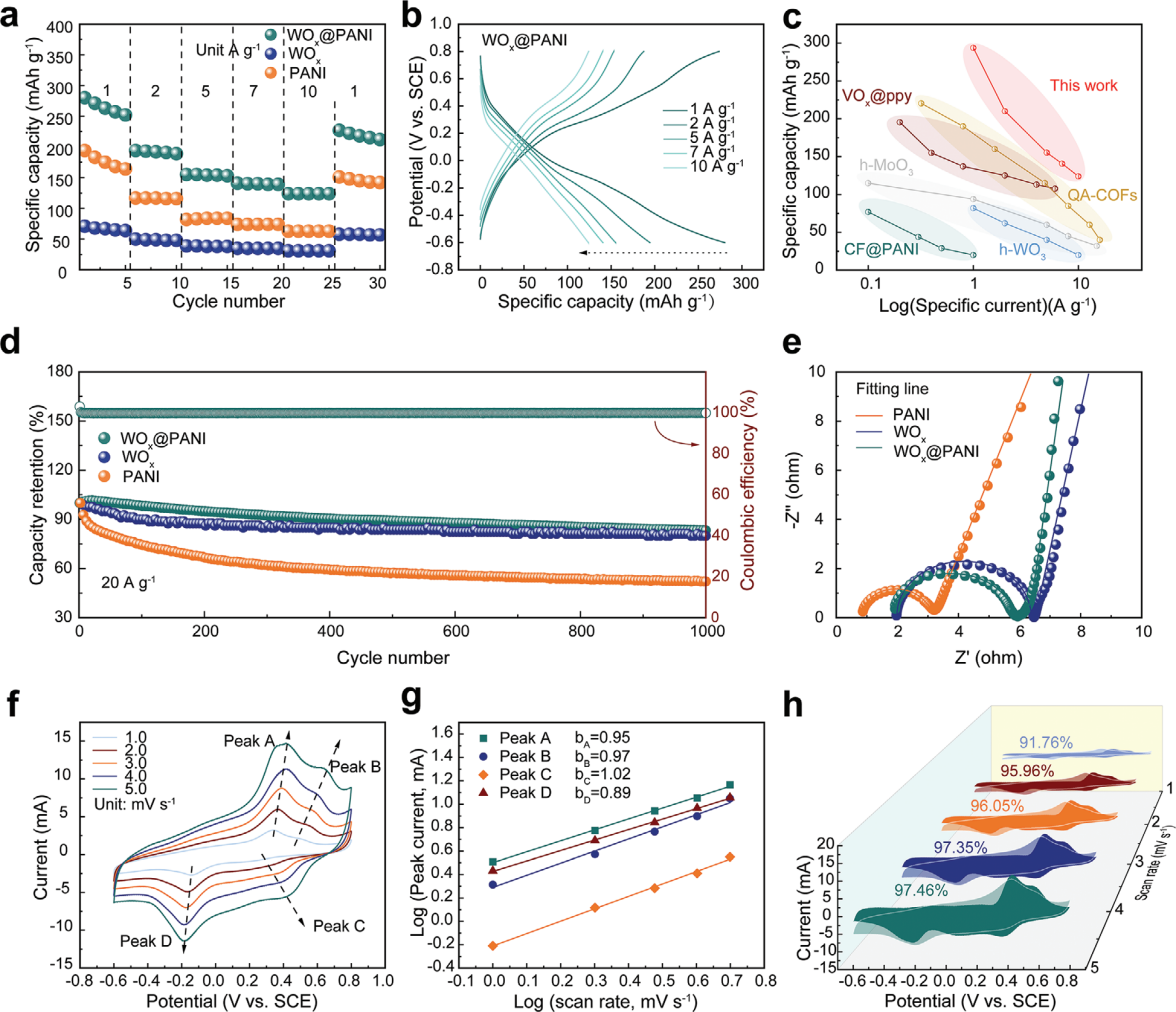
a) Rate performance of WO_x_, PANI, and WO_x_@PANI. b) GCD profiles of WO_x_@PANI at various current densities. c) Comparison of the specific capacity and specific current between WO_x_@PANI electrode and other previously reported NH_4_^+^ host materials. d) Cycle stability at 20 A g^−1^ of WO_x_@PANI, WO_x_, and PANI. e) Nyquist plots of WO_x_@PANI, WO_x_ and PANI. f) CV curves of WO_x_@PANI at various scan rates. g) The fitted *b*‐value corresponds to the four redox peaks in (f). h) Ratio of capacitive contribution at different scan rates.

The X‐ray photoelectron spectroscopy (XPS) survey spectrum of WO_x_@PANI shows clear W, O, and N signals, further confirming the successful fabrication of the composite sample (Figure S4a, Supporting Information). The N 1s spectrum of WO_x_@PANI was deconvoluted into four N components (Figure 1f): −N═ (398.04 eV), −NH− (399.18 eV), −NH^+^− (400.18 eV), and ═NH^+^− (401.45 eV).^[^ ^35^, ^36^ ^]^ Among them, the −NH− component corresponds to the oxidized state, while the other three components correspond to the reduced state. The nearly equal amounts of oxidized and reduced components indicate the emeraldine state of PANI.^[^ ^37^, ^38^ ^]^ Moreover, the high‐resolution N 1s spectrum of WO_x_@PANI show higher −NH^+^− and ═NH^+^− content, indicating a significantly higher protonation level compared to PANI (Figure S4b, Supporting Information). According to previous studies, highly protonated environments enhance the conductivity of electrode materials, thereby improving their electrochemical performance.^[^ ^39^, ^40^, ^41^, ^42^, ^43^ ^]^ The W 4f spectra of WO_x_@PANI can be fitted with four peaks (Figure 1g). The binding energies of W 4f_5/2_ and W 4f_7/2_ are located at ≈37.75 and 35.61 eV, respectively, corresponding to W^6+^. The shoulder peaks at 35.89 and 33.77 eV are attributed to W^5+^.^[^ ^44^, ^45^ ^]^ The W^6+^ content in the composites is higher than that in WO_x_, attributed to the elevated W valence state caused by the positive potential applied during the electrodeposition of PANI. Interestingly, the N 1s spectrum of the WO_x_@PANI electrode shifts to a higher energy compared to PANI, and the W 4f spectrum of the composite electrode shifts to a lower energy compared to WO_x_. This result suggests a tendency for electron transfer from PANI to WO_x_, i.e., strong coordination (N: →W^6+^) between the high‐valence state W and the lone electron pair of N in PANI.^[^ ^46^, ^47^ ^]^ The FT‐IR and XPS results indicate the coupling of multiple interfacial interactions between WO_x_ and PANI. The O atom in WO_x_ acts as a proton acceptor, and forming hydrogen bonding (N‐H···O) with the hydrogen nucleus in amine groups of PANI. Simultaneously, the lone electron pair of the N atom is strongly attracted to the metal cation W^6+^, forming a strong coordination interaction (N: →W^6+^). These two interactions are interwoven and synergistically regulate the chemical environment at the interfaces (Figure 1h). The high valence state of W in metal oxides contributes to the enhancement of the Faraday redox process. Meanwhile, the shared electron pair between N and H atoms in the polymers tilts strongly toward the N side, leaving H in an almost protonated state, which leads to an elevated level of protonation of PANI. These synergistic effects arising from interfacial interactions enhance the electrochemical performance of WO_x_@PANI (discussed in **Figure** **2**).

### 2.2 Electrochemical Properties Based on Interfacial Chemical Modifications

The electrochemical performances of WO_x_, PANI, and WO_x_@PANI electrodes were systematically investigated in a three‐electrode cell with a 0.5 m (NH_4_)_2_SO_4_ electrolyte. The cyclic voltammetry (CV) curves of the electrodes were first collected at 5 mV s^−1^, and the WO_x_@PANI displayed richer redox peaks and higher electrochemical activity (Figure S5, Supporting Information). The CV curves exhibit results consistent with the discharge capacity of the three electrodes at various current densities (Figure 2a; Figure S6, Supporting Information). Figure 2b displays the galvanostatic charge/discharge (GCD) curves of WO_x_@PANI at different current densities. The WO_x_@PANI electrode displayed a high specific capacity of 280.3 mAh g^−1^ at 1 A g^−1^ and maintained 123.9 mAh g^−1^ even when the current density increased to 10 A g^−1^, indicating good rate performance. Notably, these values are remarkably superior to most NH_4_^+^ host materials that have been reported to date, as illustrated in Figure 2c, such as h‐MoO_3_ (≈115 mAh g^−1^ at 0.1 A g^−1^),^[^ ^13^ ^]^ covalent organic framework (QA‐COF) (≈220.4 mAh g^−1^ at 0.5A g^−1^),^[^ ^15^ ^]^ VO_x_@PPy (≈195.4 mAh g^−1^ at 0.2 A g^−1^),^[^ ^16^ ^]^ and h‐WO_3_ (≈82 mAh g^−1^ at 1 A g^−1^)^[^ ^17^ ^]^ (detailed comparisons are summarized in Table S1, Supporting Information). We also obtained the GCD curve of the functionalized carbon cloth (FCC) substrate. The FCC substrate exhibits an areal capacity of only 0.0366 mAh cm^−2^ at a current density of 2.23 mA cm^−2^, which corresponds to 5.85% of the total WO_x_@PANI capacity (Figure S7, Supporting Information). Therefore, the electrochemical activity of the composite electrode is primarily due to the active materials, not the substrate. In addition to its ultrahigh capacity, the composite electrode also demonstrates excellent cycling stability. As shown in Figure 2d, the pure PANI exhibited significant capacity degradation (52% capacity retention after 1000 cycles), which is attributed to the inevitable large volume changes of the conductive polymer during charging/discharging, which leads to its dissolution in aqueous electrolyte. After integration with metal oxide, WO_x_@PANI electrode exhibited excellent cycling stability, retaining 83% of its capacity after 1000 cycles at 20 A g^−1^ and 72% after 5000 cycles at the same current density (Figure S8, Supporting Information). Generally, the capacity fluctuations in the long cycle test of electrode materials are related to the alterations of the thermodynamic state of the electrolyte, which should be attributed to the increase in temperature in the test room.^[^ ^48^, ^49^, ^50^ ^]^ The improvement in electrochemical stability is attributed to the metal oxides acting as mechanical supports for the polymers, thereby controlling their volume changes to some extent. This has been demonstrated in numerous previous studies.^[^ ^51^, ^52^ ^]^ In order to support the structural stability, SEM of WO_x_@PANI composite electrode after high‐current cycling was performed. As shown in Figure S9 (Supporting Information), although partial cracks inevitably appeared on the surface of the WO_x_@PANI composite after cycling, the composite still maintained its original cauliflower‐like morphology and was tightly wrapped around the carbon cloth fibers. This result visually demonstrates the excellent structural stability of WO_x_@PANI under high‐current cycling. Figure 2e shows the electrochemical impedance spectra (EIS) of the three electrodes. Generally, the high‐frequency region of the impedance curve reflects charge transfer kinetics, while the low‐frequency region is related to ion diffusion kinetics.^[^ ^53^, ^54^, ^55^ ^]^ The charge transfer resistance (*R*_ct_) associated with the semicircles in the high‐frequency region of WO_x_@PANI is significantly smaller than that of WO_x_, indicating that it has a more conductive property for fast charge transfer. Since the slope in the low‐frequency region is negatively related to the ion diffusion resistance, WO_x_@PANI has the lowest NH_4_^+^ diffusion resistance among the three electrodes (Table S2, Supporting Information; Figure 2e; and Figure S10, Supporting Information).

To study the reaction kinetics of WO_x_@PANI electrode, the CV curves at various scan rates from 1.0 to 5.0 mV s^−1^ were recorded (Figure 2f). The dependency of peak current (*i*) on scan rate (*ν*) is in accordance with the equation: *i* = *aν^b^*, where both variables *a* and *b* are adjustable (*b* varies from 0.5 to 1.0). In principle, a *b*‐value approaching 0.5 represents diffusion‐dominated electrochemical processes, while a *b*‐value approaching 1.0 is indicative of pseudocapacitive behavior. Four distinct peaks (peaks A–D) were observed in Figure 2f and the *b*‐values were fitted to be 0.95, 0.97, 1.02, and 0.89, respectively, indicating that the energy storage process of WO_x_@PANI is predominated by the pseudocapacitive redox reactions (Figure 2g). Besides, the capacity contribution of the WO_x_@PANI was quantified using the equation: *i*(V) = *k*_1_ *ν* + *k*_2_ *ν* ^1/2^, where the current *i* at the specific potential (V) can be decomposed into the capacitive‐controlled part *k*_1_ *ν* and diffusion‐controlled part *k*_2_ *ν* ^1/2^, respectively. As summarized in Figure 2h, the contribution ratio of capacitive‐controlled increases with the scan rate, and accounting for ≈97.46% of the total capacity at 5 mV s^−1^, further demonstrating the faster diffusion kinetics of the WO_x_@PANI electrode dominated by the capacitive process.

### 2.3 Enhanced NH_4_^+^/H^+^ Co‐Insertion Mechanism Based on Interfacial Chemistry

**Figure 3:**
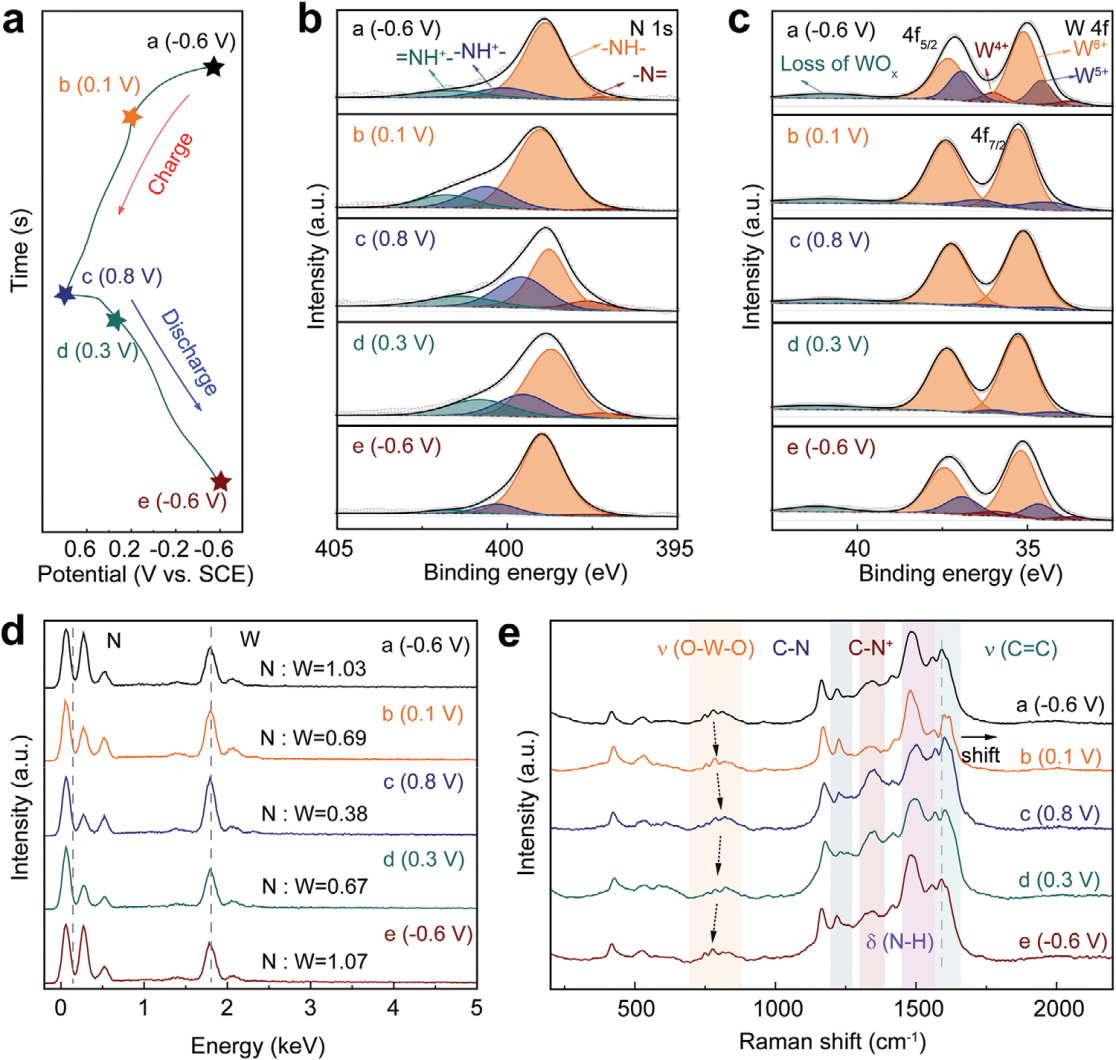
a) The second GCD curve of WO_x_@PANI at 1 A g^−1^ in 0.5 m (NH_4_)_2_SO_4_ aqueous electrolyte. b) N 1s XPS spectra, c) W 4f XPS spectra, d) EDX spectra, and e) Raman spectra corresponded to various states labeled in (a).

To explore the charge storage mechanism of WO_x_@PANI in NH_4_^+^ aqueous electrolyte, various ex situ characterizations were carried out at selected states of GCD curve (**Figure** **3**a). Figure 3b shows the ex situ XPS spectra of N 1s for the WO_x_@PANI during the charging/discharging process. During the charging process (from state a to state c), the intensity of −N═ (398.04 eV), −NH^+^− (400.18 eV), and ═NH^+^− (401.45 eV) increases, while the intensity of the −NH− component decreases, indicating the conversion of PANI from the reduced state to the oxidized state. During the discharging process (from state c to state e), the trend is reversed. Notably, the significant increase in −NH− species during discharge should be the joint effect of the inserted NH_4_^+^ and the transition of PANI to the reduced state.^[^ ^56^ ^]^ The W 4f spectra of WO_x_@PANI at different states during charging/discharging are shown in Figure 3c. During discharge, the content of W^5+^ gradually increases and W^4+^ species can be detected at a potential of −0.6 V, indicating that the insertion of NH_4_^+^ leads to a gradual decrease in the average valence of W. In contrast, the valence evolution of W during charge is completely opposite, confirming the highly reversible insertion/desertion process of NH_4_^+^.Additionally, the ex situ energy dispersive X‐ray spectroscopy (EDX) spectra demonstrate a reversible decrease/increase in the N/W ratio during charging/discharging (from states a to states e), further illustrating the NH_4_^+^ storage mechanism of the WO_x_@PANI composite electrode (Figure 3d). This result is further supported by Raman analysis at different charge states (Figure 3e). The signal located at 778 cm^−1^ is attributed to the O‐W‐O stretching vibration.^[^ ^17^, ^57^ ^]^ The intensity of the this signal is weakened during the discharge process due to the decrease in the valence state of W after the insertion of NH_4_^+^, leading to a redshift of the corresponding peak. In addition, the characteristic band of the quinoid segments in PANI at 1592 cm^−1^ (ν C═C) shifts significantly and gradually returns to its original position, indicating the high reversibility of the charging and discharging process. Changes in the band intensity at 1220 cm^−1^ (C‐N) and 1340 cm^−1^ (C‐N^+^) correspond to the transition of PANI between the oxidized and reduced states.^[^ ^58^, ^59^ ^]^ Interestingly, the band intensity of the imine nitrogen at ≈1484 cm^−1^ (C═N) decreases to a minimum in the fully oxidized state (0.8 V) and increases to a maximum when discharged to −0.6 V. This result can be explained by the interference of NH_4_^+^ related vibrational signals with similar spectral peaks.^[^ ^60^ ^]^ To eliminate the influence of NH_4_^+^ stored by WO_x_ in the composite sample on the analysis, we collected Raman spectra of the pure PANI electrode at different charging and discharging states (Figure S11, Supporting Information). Notably, the band intensity at 1484 cm^−1^ (C = N) gradually increases during charging, and a shoulder band emerges at 1490 cm^−1^ when charging to 0.1 V, indicating that the H^+^ (1476 cm^−1^) and NH_4_^+^ (1490 cm^−1^) are coordinated with the imine nitrogen of the semioxidized state PANI (emeraldine state, EB). However, when discharged to 0.3 V, the disappearance of the additional shoulder band indicates that protons have replaced the ammonium ions on the imine nitrogen site. The ex situ N 1s spectra of pure PANI electrode also reveal a unique change in nitrogen intensity during charging and discharging, associated with the H^+^ substitution reaction (Figure S12, Supporting Information). Specifically, the nitrogen intensity at 0.1 V is almost the equal of that at 0.8 V during charging, while the strength drops to a minimum at 0.3 V during discharging, and gradually increases to a maximum when discharged to −0.6 V. In other words, during the process of charging from the fully reduced state to the fully oxidized state, followed by discharging to the semioxidized state, the ammonium ions are gradually decoordinated from PANI until their content reaches a minimum level in the semioxidization state, which corresponds to the initiation of the H^+^ substitution reaction.

**Figure 4:**
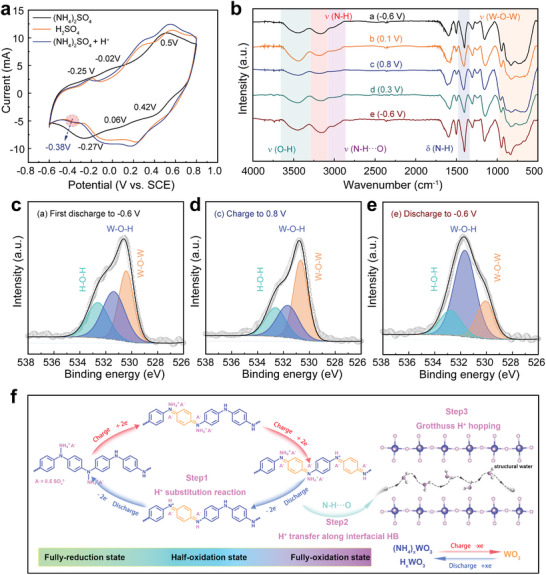
a) CV curves of the WO_x_@PANI electrode in 0.05 m (NH_4_)_2_SO_4_ (pH 5.7), 0.05 m H_2_SO_4_ (pH 1.2), and 0.05 m (NH_4_)_2_SO_4_ at pH 1.2 by adding H_2_SO_4_ electrolytes in a three‐electrode cell at 5 mV s^−1^. b) FT‐IR spectra of WO_x_@PANI at various charging/discharging states. c–e) O 1s XPS spectra of pure WO_x_ electrode at selected states. f) Schematic diagram of the charge storage mechanism of WO_x_@PANI.

In general, the aqueous energy storage process involves the co‐insertion of H^+^ with primary carriers, exhibiting rapid proton transfer through the Grotthuss mechanism.^[^ ^11^, ^61^, ^62^, ^63^, ^64^, ^65^ ^]^ The rapid migration of protons through a hydrogen bonding chain is analogous to Newton's cradle, where long‐distance transport is achieved through local displacement. Previous studies have demonstrated that Grotthuss conduction can be triggered in both electrolytes and transition metal oxide electrode materials with layered, tunnel, or open‐framework structures in a hydrogen‐bonding environment.^[^ ^66^, ^67^, ^68^, ^69^ ^]^ To study this, we firstly analyzed various CV curves obtained for WO_x_@PANI in 0.05 m (NH_4_)_2_SO_4_ (pH 5.7), 0.05 m H_2_SO_4_ (pH 1.2), and 0.05 m (NH_4_)_2_SO_4_ adjusted to pH 1.2 by adding H_2_SO_4_ (**Figure** **4**a). The use of a low‐concentration electrolyte of 0.05 m (NH_4_)_2_SO_4_ aimed to eliminate the effect of proton production from the hydrolysis of (NH_4_)_2_SO_4_. Specifically, in 0.05 m (NH_4_)_2_SO_4_ electrolyte, two oxidation peaks at ≈0.5/−0.02 V versus SCE and two reduction peaks at 0.42/0.06 V versus SCE during sweep process correspond to the conversion of PANI from the fully oxidized state (pernigraniline salt, PNS) to the fully reduced state (leucoemeraldine, LE). Meanwhile, the oxidation peak at ≈−0.25 V versus SCE and the reduction peak at −0.27 V versus SCE correspond to the NH_4_^+^ storage process of WO_x_. However, in the 0.05 m H_2_SO_4_ electrolyte, the positions of the above peaks have shifted. Interestingly, a new reduction peak is observed at ≈−0.38 V versus SCE in both 0.05 m H_2_SO_4_ and 0.05 m (NH_4_)_2_SO_4_ adjusted to pH 1.2 with H_2_SO_4_, indicating a new reduction process occurs during discharge. This new peak at ≈−0.38 V in both electrolytes can be inferred to correspond to the reaction between H^+^ and the composite electrode. In order to quantify the proportion of capacity contribution of H^+^ during the discharge process, we further investigated and compared the electrochemical behaviors of WO_x_@PANI in 0.5 m (NH_4_)_2_SO_4_ (pH = 4.63) and dilute H_2_SO_4_ (pH 4.63) electrolyte.^[^ ^70^, ^71^, ^72^ ^]^ As shown in Figure S13 (Supporting Information), Comparing the integral area results of the CV curves in the two cases, we found that the maximum capacity proportion of H^+^ from WO_x_@PANI in 0.5 m (NH_4_)_2_SO_4_ electrolyte is 16.8%, which further evidence for the interaction of H^+^ with the host material. Actually, when H^+^ is inserted in the electrode material, the pH of the electrolyte will increase, even if the difference is very subtle. To demonstrate this, we designed an in situ pH detection based on a Swagelok cell. Considering that the pH change is more significant at the electrode‐electrolyte interface, Swagelok cell was selected to amplify this effect by reducing the amount of electrolyte. As shown in Figure S14a (Supporting Information), the pH of the electrolyte exhibits a gradual increase with repeated charging and discharging. Figure S14b (Supporting Information) clearly records the pH detection device and the pH of the electrolyte at different times during the cycling process. After 36 min, the pH of the electrolyte increased from 4.65 at the initial stage to 5.15, which indicates that a portion of H^+^ in the electrolyte is inserted in the electrode material.

So as to study the NH_4_^+^/H^+^ co‐insertion mechanism in more detail, the ex situ FT‐IR spectra of the WO_x_@PANI in a 0.5 m (NH_4_)_2_SO_4_ electrolyte were first performed (Figure 4b). The absorption band at 1402 cm^−1^ corresponds to the bending vibration of N‐H. We observed a periodic increase/decrease of the N‐H signal during charging/discharging, demonstrating that the ammonium ion undergoes a reversible insertion/desertion process in WO_x_@PANI (Figure S15, Supporting Information). Moreover, the two absorption peaks at 3014 cm^−1^ (N‐H···O stretching method) and 3170 cm^−1^ (N‐H stretching method) continuously weakened during charging and gradually increased during subsequent discharging, further illustrating the hydrogen bonding storage mechanism of the ammonium ions with the host material.^[^ ^14^, ^73^ ^]^ Notably, the enhancement of the absorption peak at 3442 cm^−1^ during discharging, corresponding to the vibration of O‐H, suggests that H^+^ is also involved in the energy storage process.^[^ ^54^, ^74^ ^]^ As shown in Figure 4c–e, the O 1s XPS spectra of the pure WO_x_ in a 0.5 m (NH_4_)_2_SO_4_ electrolyte at three charging and discharging states were collected. During charging, the content of W‐O‐W (530.4 eV) increased, while the content of the O‐related component (W‐O‐H) at 531.6 eV decreased (form state a to state c).^[^ ^44^, ^75^ ^]^ When discharged again to −0.6 V, the peak intensity of W‐O‐W decreased to a minimum, while that of W‐O‐H increased to a maximum (from state c to state e). This result, combined with the ex situ Raman analysis (discussed in Figure 3), corroborates the mechanism of H^+^ storage in composites. This mechanism can be summarized as the occurrence of proton substitution reactions within the polymer module and reversible W‐O‐W/W‐O‐H transformation within the metal oxide module during charging/discharging. The proposed NH_4_^+^/H^+^ co‐insertion mechanism during charging and discharging is illustrated in Figure 4f. The WO_x_@PANI composite electrode exhibits a hybrid charge storage mechanism co‐dominated by conversion reactions and ion insertion processes. Specifically, PANI undergoes a conversion from fully reduced state to half‐oxidized state to fully oxidized state, and a reversible redox reaction occurs between tungsten oxide and tungsten bronze (H_x_WO_3_ or (NH_4_)_x_WO_3_). As the conversion reaction proceeds, NH_4_^+^ continuously (de)coordinates with nitrogen sites in PANI and is reversibly inserted/deserted in the WO_x_ interlayer accompanied by the formation/breaking of hydrogen bonds. Notably, H^+^ undergoes a continuous three‐step energy storage process. First, during the conversion of PANI from the fully oxidized state to the semioxidized state, H^+^ replaces NH_4_^+^ originally coordinated to the nitrogen site of the imine. Subsequently, the H^+^ decoordinated from the imine nitrogen sites can rapidly diffuse into WO_x_ through the hydrogen bonding interactions (N‐H···O) between the interfaces. Finally, the interlayer water molecules in WO_x_ act as a channel for H^+^ hopping, activating the Grotthuss conduction mechanism and enabling rapid proton energy storage.

### 2.4 Assembly of High‐Performance Aqueous Ammonium Ion Battery

**Figure 5:**
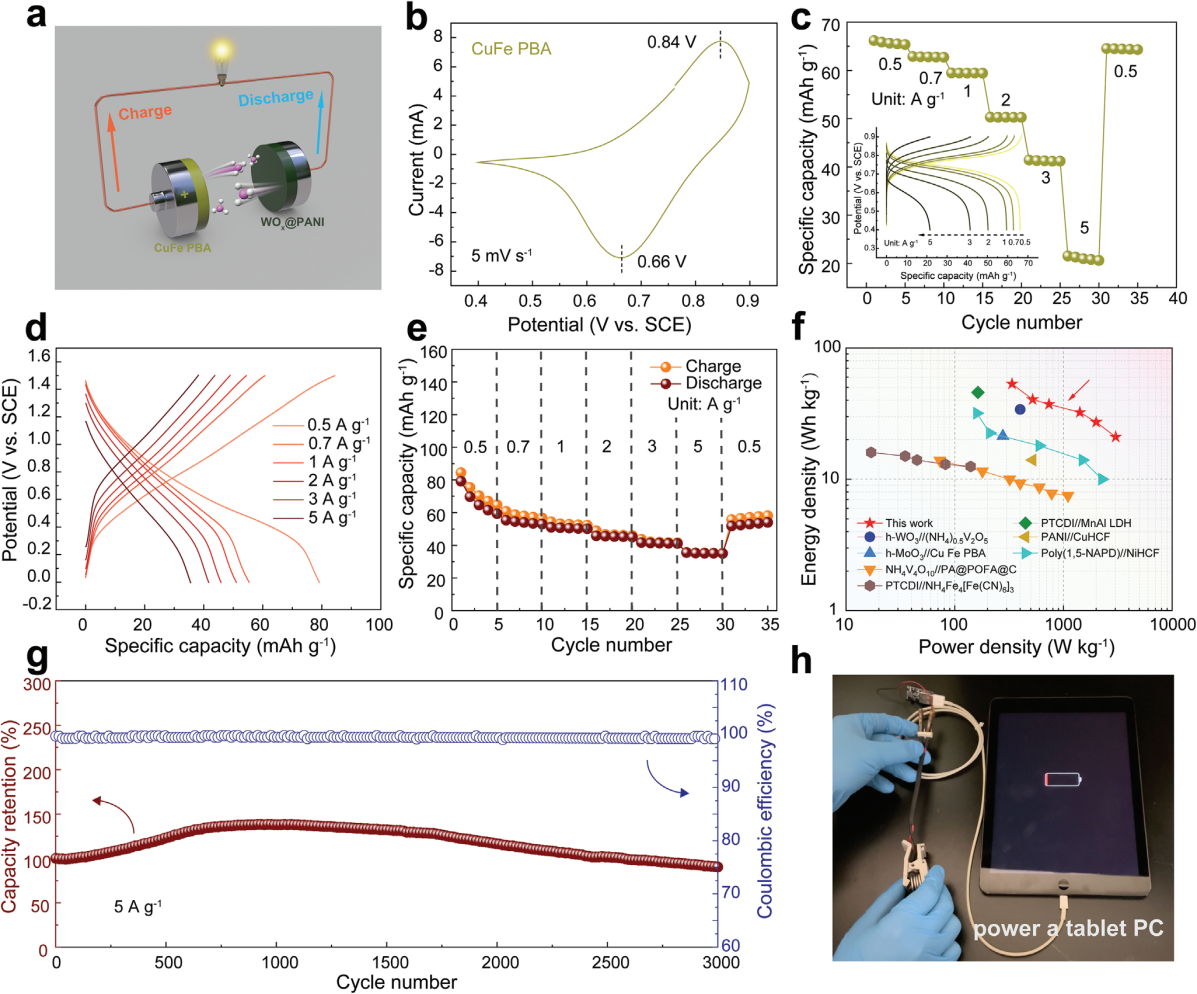
a) Schematic diagram of WO_x_@PANI//CuFe PBA rocking‐chair battery. b) CV curve at 5 mV s^−1^ and c) rate performance and GCD curves (inset) of the CuFe PBA cathode. d) GCD curves at different current density. e) Rate performance. f) The Ragone plots. g) Long‐term stability at 5 A g^−1^ of the WO_x_@PANI//CuFe PBA device. h) Demonstration of the AAIB to power a tablet PC.

A rocking‐chair AAIB was constructed using WO_x_@PANI as the anode and CuFe Prussian blue analog (PBA) as the cathode. **Figure** **5**a illustrates the schematic diagram of WO_x_@PANI//CuFe PBA rocking‐chair battery, with NH_4_^+^ ions in the electrolyte shutting back and forth between the anode and cathode. SEM images show that CuFe PBA consists of many irregular clusters of cubic nanoparticles (Figure S16, Supporting Information). The diffraction peaks in the XRD pattern (Figure S17, Supporting Information) are well indexed to Cu[Fe(CN)_6_]_0.667_. This indicates that the as‐prepared CuFe PBA is highly crystalline. The CV curve of CuFe PBA at 5 mV s^−1^ (Figure 5b) shows an oxidation peak at 0.84 V and a corresponding reduction peak at 0.66 V, indicating its high redox potential. The GCD curves of CuFe PBA show a specific capacity of 66.2 mAh g^−1^ at 0.5 A g^−1^, and after a series of charging and discharging cycles at different current densities, the specific capacity remains at 64.6 mAh g^−1^ at 0.5 A g^−1^, demonstrating its outstanding electrochemical stability (Figure 5c). After pairing with the CuFe PBA cathode, the full battery displayed a capacity of 79.2 mAh g^−1^ at 0.5 A g^−1^ and 35.6 mAh g^−1^ at 5 A g^−1^ (based on the total active mass of cathode and anode materials with mass ratio of 3:1), demonstrating good rate capability (Figure 5d,e). It also shows ≈100% coulombic efficiency during cycling and good cycle stability with a capacity retention rate of 90.3% after 3000 cycles at 5 A g^−1^ (Figure 5g). The capacity rise phase observed in the long cycle test can be attributed to the improved wettability of the electrode interface.^[^ ^76^ ^]^ Remarkably, the full cell exhibits a high energy density of 53.3 Wh kg^−1^ at the power density of 336.9 W kg^−1^ and retains 21.0 Wh kg^−1^ at 3024.0 W kg^−1^ (based on the total active mass of cathode and anode), exceeding most previously reported aqueous NH_4_^+^ devices (Figure 5f).^[^ ^13^, ^17^, ^53^, ^77^, ^78^, ^79^, ^80^ ^]^ Finally, we assembled and connected four WO_x_@PANI//CuFe PBA batteries in a 2032‐type coin configuration in series to successfully power a tablet PC, further demonstrating the feasibility of practical application (Figure 5h; Video S1, Supporting Information).

## 3 Conclusion

In summary, we fabricated a WO_x_@PANI composite electrode for aqueous ammonium ion batteries using a two‐step electrodeposition method on a functionalized carbon cloth substrate. The coupling of multiple interfacial interactions between WO_x_ and PANI enhances charge storage behavior, effectively increasing the protonation level of PANI and affects the coordination environment of tungsten metal atoms. This, thereby, improves the electrical conductivity and stability of the composite. Therefore, the WO_x_@PANI electrode delivers an ultrahigh specific capacity of 280.3 mAh g^−1^ at 1 A g^−1^ and maintains a high specific capacity of 123.9 mAh g^−1^ at 10 A g^−1^, exceeding the vast majority of reported NH_4_^+^ host materials. An NH_4_^+^/H^+^ co‐insertion mechanism in 0.5 m (NH_4_)_2_SO_4_ for the WO_x_@PANI electrode was demonstrated by various ex situ analyses. The experimental results showed that the interfacial hydrogen bonding network can effectively facilitate the Grotthuss conduction mechanism and enable the H^+^ hopping conduction between water molecules in the WO_x_ interlayer structure. In this study, the NH_4_^+^ storage performance of WO_x_@PANI is systematically investigated for the first time, with an emphasis on the effect of coupling multiple interfacial interactions on the energy storage mechanism. This research provides valuable insights for the further development of NH_4_^+^ host materials modified by interfacial chemistry.

## Conflict of Interest

The authors declare no conflict of interest.

## Supporting information

Supporting Information

Supplemental Video 1

## Data Availability

The data that support the findings of this study are available from the corresponding author on reasonable request.
