# Supplementary material for: Coordination and Hydrogen Bond Chemistry in Tungsten Oxide@Polyaniline Composite toward High‐Capacity Aqueous Ammonium Storage
Source: Small. 2024 Aug 18;20(51):2405592. doi: 10.1002/smll.202405592 (PMC11657061; doi:10.1002/smll.202405592)
Supplement: Supplementary file 1 — Supporting Information [file SMLL-20-2405592-s002.docx]

**Supplementary Information**

**Coordination and Hydrogen Bond Chemistry in** **Tungsten Oxide@Polyaniline Composite toward High-Capacity Aqueous Ammonium Storage**

*Shuai Mao ^a^, Xu Han ^c^,* *Zi-Hang Huang ^a,^*, Hui Li ^a,b^, and Tianyi Ma ^b,^**

^a^ Institute of Clean Energy Chemistry, Key Laboratory for Green Synthesis and Preparative Chemistry of Advanced Materials of Liaoning Province, College of Chemistry, Liaoning University, Shenyang 110036, China

E-mail: huangzihang@lnu.edu.cn

^b^ School of Science, RMIT University, Melbourne, VIC 3000, Australia

E-mail: tianyi.ma@rmit.edu.au

^c^ Engineering Laboratory of Advanced Energy Materials, Ningbo Institute of Materials Technology and Engineering Chinese Academy of Sciences, Ningbo 315201, China

(These authors contributed equally: Shuai Mao, Xu Han)

**1. Experimental section**

*1.1 Materials*

Sodium tungstate dihydrate (Na_2_WO_4_·2H_2_O), hydrogen peroxide (H_2_O_2_, 30%), sulfuric acid (H_2_SO_4_, 95%~98%), aniline (C_6_H_7_N), copper (Ⅱ) sulfate pentahydrate (CuSO_4_·5H_2_O) and potassium ferricyanide (K_3_[Fe(CN)_6_]) were purchased from Sinopharm Chemical Reagent Co., Ltd. All the chemical reagents were used as received, expect aniline which was used after reduced pressure distillation. Carbon cloth (CC) was purchased from Shanghai HESEN Electric Co., Ltd. (China) and graphite foils manufactured from natural expanded graphite were purchased from SGL Group (Germany).

*1.2 Functionalization of carbon cloth (FCC)*

Functionalization of carbon cloth (working area:1.0 × 1.0 cm^2^) was carried out in a three-electrode system using a saturated calomel electrode (SCE) as a reference electrode and a piece of graphite foil (1.5 × 2.0 cm^2^) as the counter electrode, respectively. A constant potential of 1.85 V vs. SCE was first applied to the working electrode (carbon cloth) for 30 min with 0.5 M KNO_3_ as the electrolyte to improve the wettability of the electrode. The treated electrode was then further scanned using cyclic voltammetry for 50 cycles at the scan rate of 100 mV s^-1^ from -1 to 1 V vs. SCE using 3 M KCl as the electrolyte to restore the conductivity of the carbon cloth. The electrochemically functionalized carbon cloth was denoted as FCC.

*1.3 Electrochemical deposition of WO_x_@PANI*

The electrodeposition of all the materials was conducted in a three-electrode system, which is used SCE as the reference electrode and graphite foil as the counter electrode. Firstly, 8 mmol sodium tungstate (Na_2_WO_4_·2H_2_O) was dissolved in 100 mL of deionized water, and 0.8 mL of H_2_SO_4_ (98%) was added to acidify the solution, which was stirred at 60 °C for 10 min (the purpose of which was to enhance the acidification effect), and then 1.0 mL of H_2_O_2_ (30%) was added to obtain the peroxytungstic acid (PTA) solution was prepared. Using FCC as the working electrode and PTA as the electrolyte, WO_x_ was uniformly deposited on FCC by electrodeposition using a constant potential of -0.5 V for 3 min. The electrodes were then electrodeposited in the aqueous solution containing 0.25 M aniline and 0.5 M H_2_SO_4_ at a constant potential of 1.8 V for 1 min, and the electrodes obtained were named WO_x_@PANI. The active loadings of the electrodes were limited to be around 2.5 mg cm^-2^.

*1.4 Synthesis of Cu Fe PBA*

CuFe PBA was synthesized using a simple co-precipitation method previously reported.^[1]^ Briefly, 5 g of copper sulfate pentahydrate (CuSO_4_·5H_2_O) and 3.3 g of potassium ferricyanide (K_3_[Fe(CN)_6_]) were dissolved in 50 mL of deionized water, respectively. The copper sulfate solution was then slowly dripped into the potassium ferricyanide solution, and the mixed solution was magnetically stirred at 50 °C for 1 h. Finally, the resulting precipitates were washed several times with deionized water and ethanol, and then dried under vacuum at 60 °C overnight.

*1.5 Characterization*

XRD patterns were obtained using a Bruker D8 ADVANCE system with Cu Kα radiation source (60.0 kV, 80.0 mA) in the range of 10° to 80°. The morphology of the electrodes was characterized by a scanning electron microscope (SEM) equipped with an energy dispersive X-ray spectroscopy (EDX) detector (HITACHI, SU8010, Japan). Transmission electron microscope (TEM) images were carried out by JEM-2010. The chemical composition of the electrodes was studied by X-ray photoelectron spectroscopy (ESCALAB 250Xi, Thermo Scientific Escalab, USA) with Al Kα radiation (8.34 Å) as the excitation source. Raman spectroscopy and transmission FTIR spectra were collected using a RENISHAW inVia Raman microscope and a Thermo Nicolet 5700 spectrometer, respectively. The mass loading of active materials was measured by the weight difference of the electrode before and after electrodepositing, using a Sartorius BT25S semi-microbalance with a sensitivity of 0.01 mg.

*1.6 Electrochemical Measurements*

Electrochemical measurements of the single electrode were conducted in a three-electrode system, where modified with active materials was used as working electrode, saturated calomel electrode (SCE) as the reference electrode and graphite foil as the counter electrode, respectively. The full cell properties were measured in a 2032-type coin, where WO_x_@PANI was used as the anode, CuFe PBA was used as the cathode at a mass ratio of 2:3, Whatman GF/D glass fiber was used as the separator, and 0.5 M (NH_4_)_2_SO_4_ was used as an aqueous electrolyte, respectively. All CV tests were carried out on a CHI760 electrochemical workstation, while GCD measurements of single electrode were studied by a multichannel working station (VMP3, Bio-Logic, France) and GCD measurements of full cell were performed on the Neware battery test systems. The energy density (E) was determined based on the following equation: $E=\int_{0}^{Q} V\left( q \right)dq$, where Q is the specific capacity of cell, V is the voltage of cell, q denotes the state of discharge, and V(q) is the voltage of the cell at the state of discharge q. The corresponding power density (P) was calculated using the following equation: P = E/t, where t represents the working time, and E is the energy density.

**2. Supplementary Figures and Tables**

**
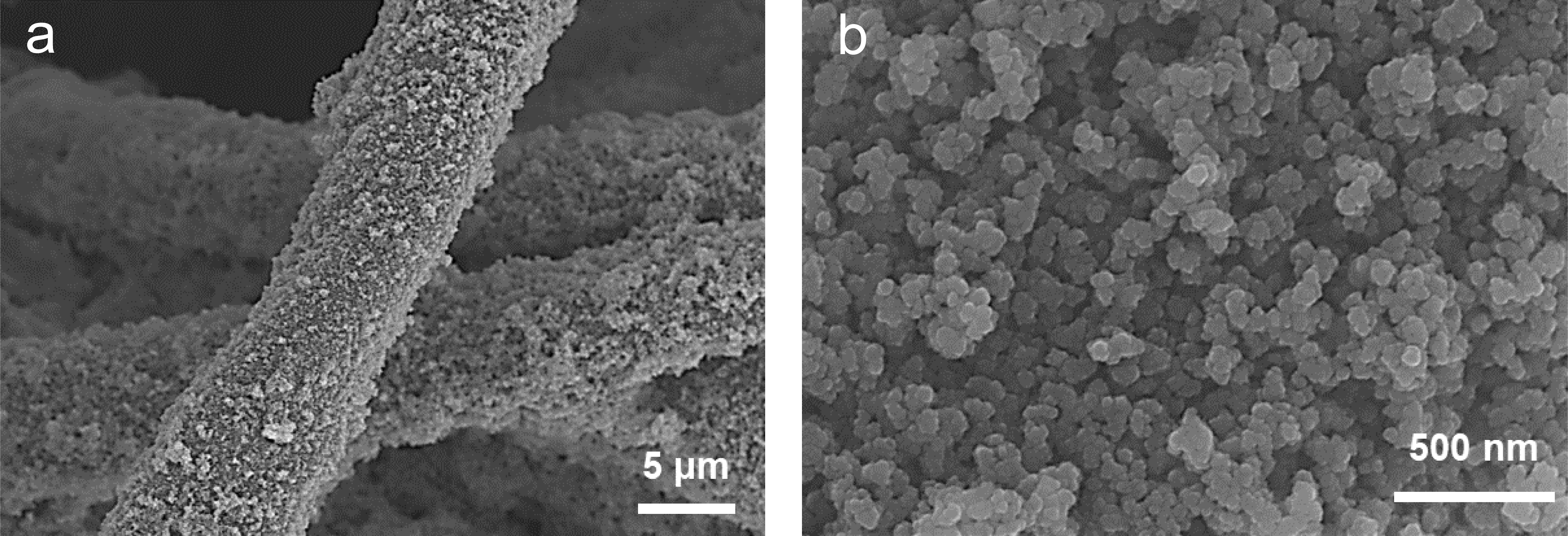
**

**Figure S1.** SEM images of WO_x_.


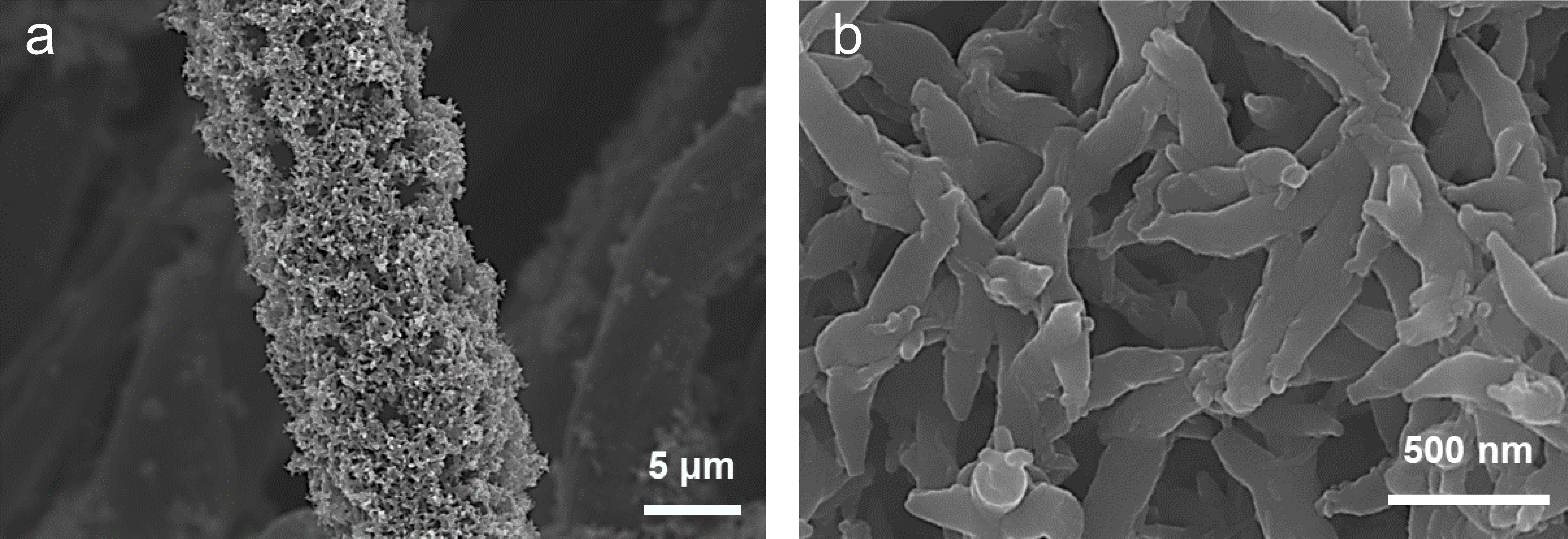


**Figure S2.** SEM images of PANI.


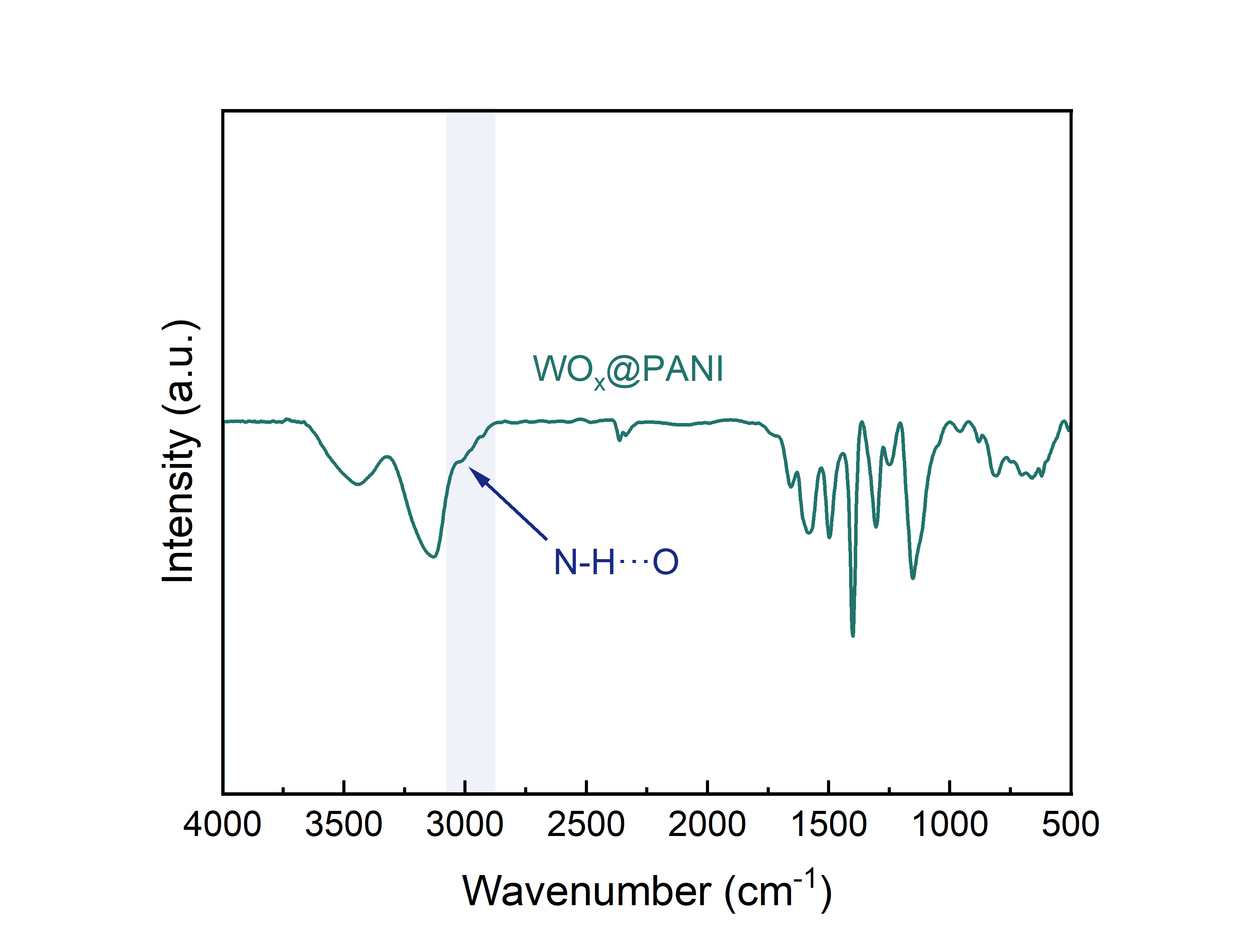


**Figure S3.** FT-IR spectra of WO_x_@PANI (wavenumber ranges 500-4000 cm^-1^).


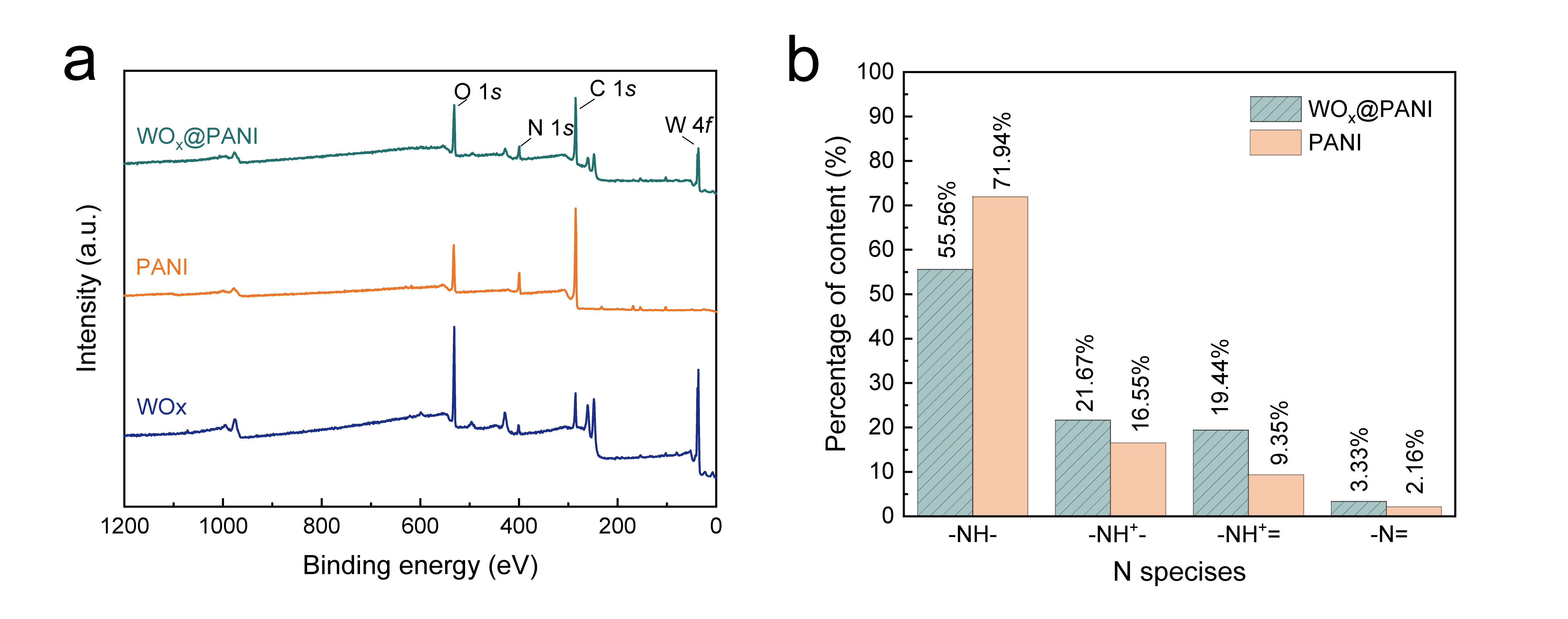


**Figure S4.** (a) XPS spectra of WO_x_@PANI, WO_x_ and PANI. (b) The percentages of the N species of WO_x_@PANI and PANI.


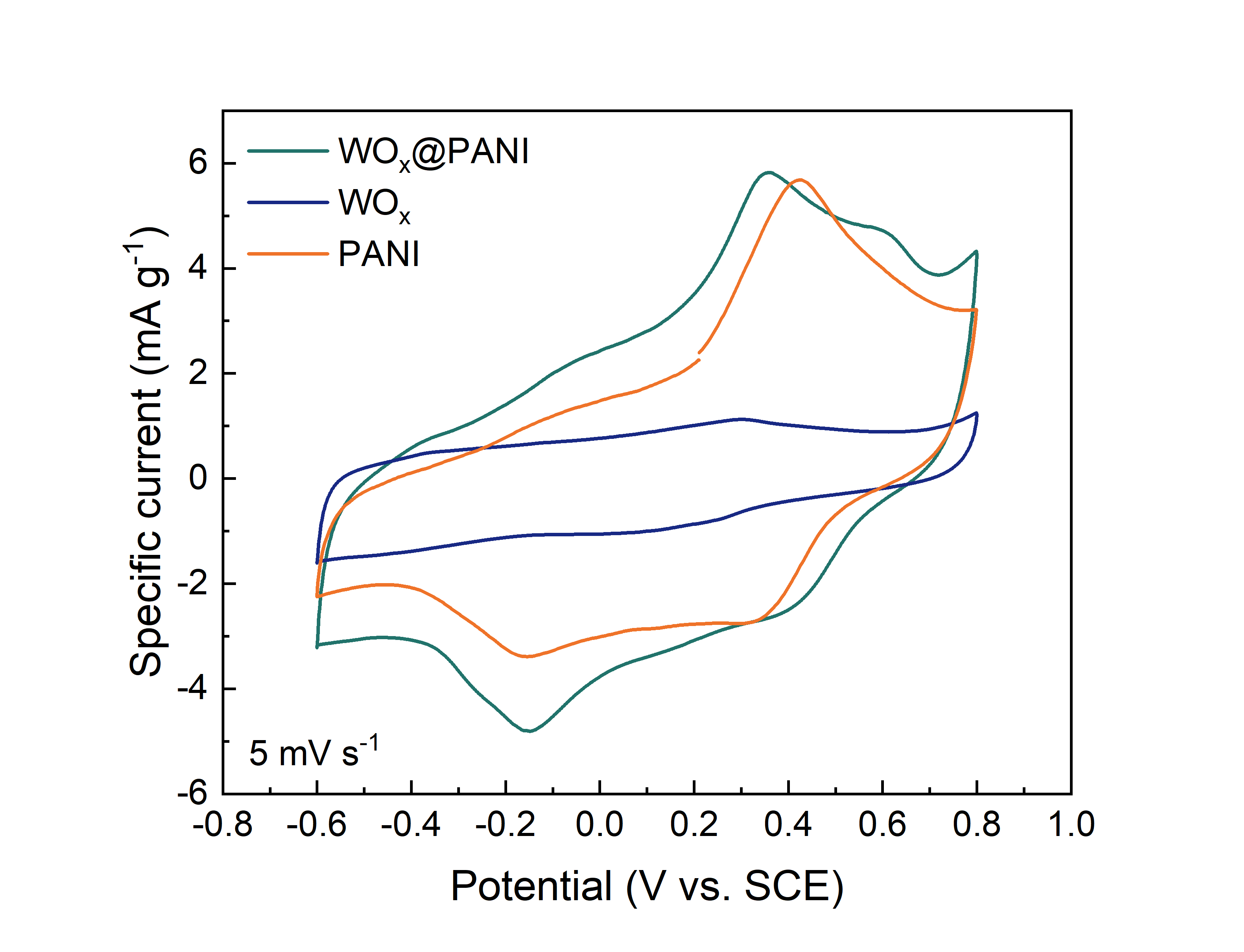


**Figure S5.** CV curves of WO_x_@PANI, WO_x_ and PANI.


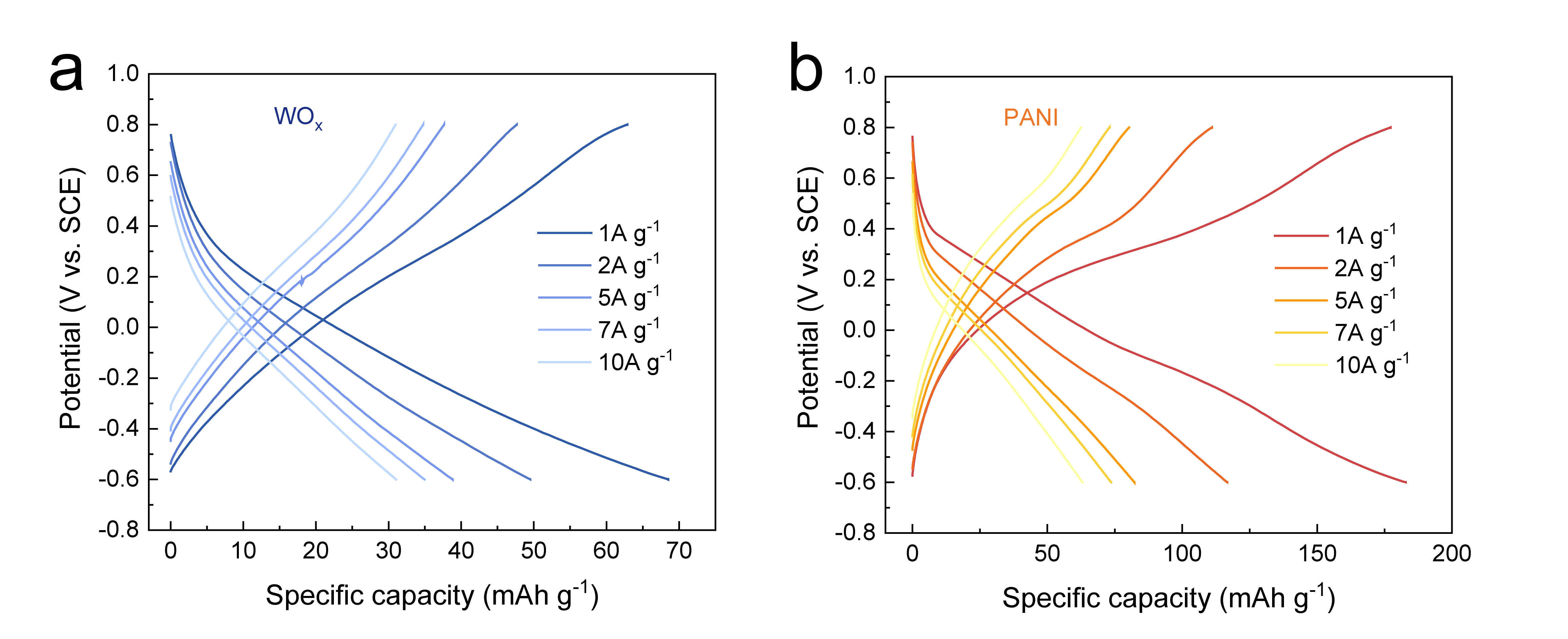


**Figure S6.** Charge/discharge curves of (a) WO_x_@PANI and (b) WO_x_.

**Table S1.** Summary of the electrode materials and corresponding electrochemical properties of AAIBs.

| *Electrode materials* | *Electrolyte* | *Capacity* | | *Cyclic performance* | *Ref.* |
| --- | --- | --- | --- | --- | --- |
| QA-COF | 0.5 M (NH_4_)_2_SO_4_ | | 220.4 mAh g^-1^ at 0.5 A g^-1^ | 80% after 7000 cycles, 6 A g^-1^ | ^[2]^ |
| VO_x_@ppy | 0.5 M NH_4_Ac | | 195.4 mAh g^-1^ at 0.2 A g^-1^ | 85% after 2000 cycles, 1 A g^-1^ | ^[3]^ |
| h-MoO_3_ | 1 M NH_4_Cl | | 115 mAh g^-1^ at 0.1 A g^-1^ | 94% after 100000 cycles, 15 A g^-1^ | ^[4]^ |
| MnO_x_ | 0.5 M NH_4_Ac | | 176 mAh g^-1^ at 0.5 A g^-1^ | 94.7% after 10000 cycles, 5 A g^-1^ | ^[5]^ |
| Berlin green | 0.5 M (NH_4_)_2_SO_4_ | | 91.5 mAh g^-1^ at 0.1 A g^-1^ | 88% after 50000 cycles, 5 A g^-1^ | ^[6]^ |
| Alloxazine | 1 M (NH_4_)_2_SO_4_ | | 138.6 mAh g^-1^ at 1 A g^-1^ | 80% after 1500 cycles, 10 A g^-1^ | ^[7]^ |
| h-WO_3_ | 1 M (NH_4_)_2_SO_4_ | | 82 mAh g^-1^ at 1 A g^-1^ | 80% after 100000 cycles, 20 A g^-1^ | ^[8]^ |
| CF@PANI | 1 M (NH_4_)_2_SO_4_ | | 77 mAh g^-1^ at 0.1 A g^-1^ | 81.8% after 100 cycles, 0.1 A g^-1^ | ^[9]^ |
| WO_x_@PANI | **0.5 M (NH_4_)_2_SO_4_** | | **280.3 mAh g^-1^ at 1 A g^-1^** | **83% after 1000 cycles, 20 A g^-1^** | **This work** |
|  |  |  | **123.9 mAh g^-1^ at 10 A g^-1^** | **72% after 5000 cycles, 20 A g^-1^** |  |


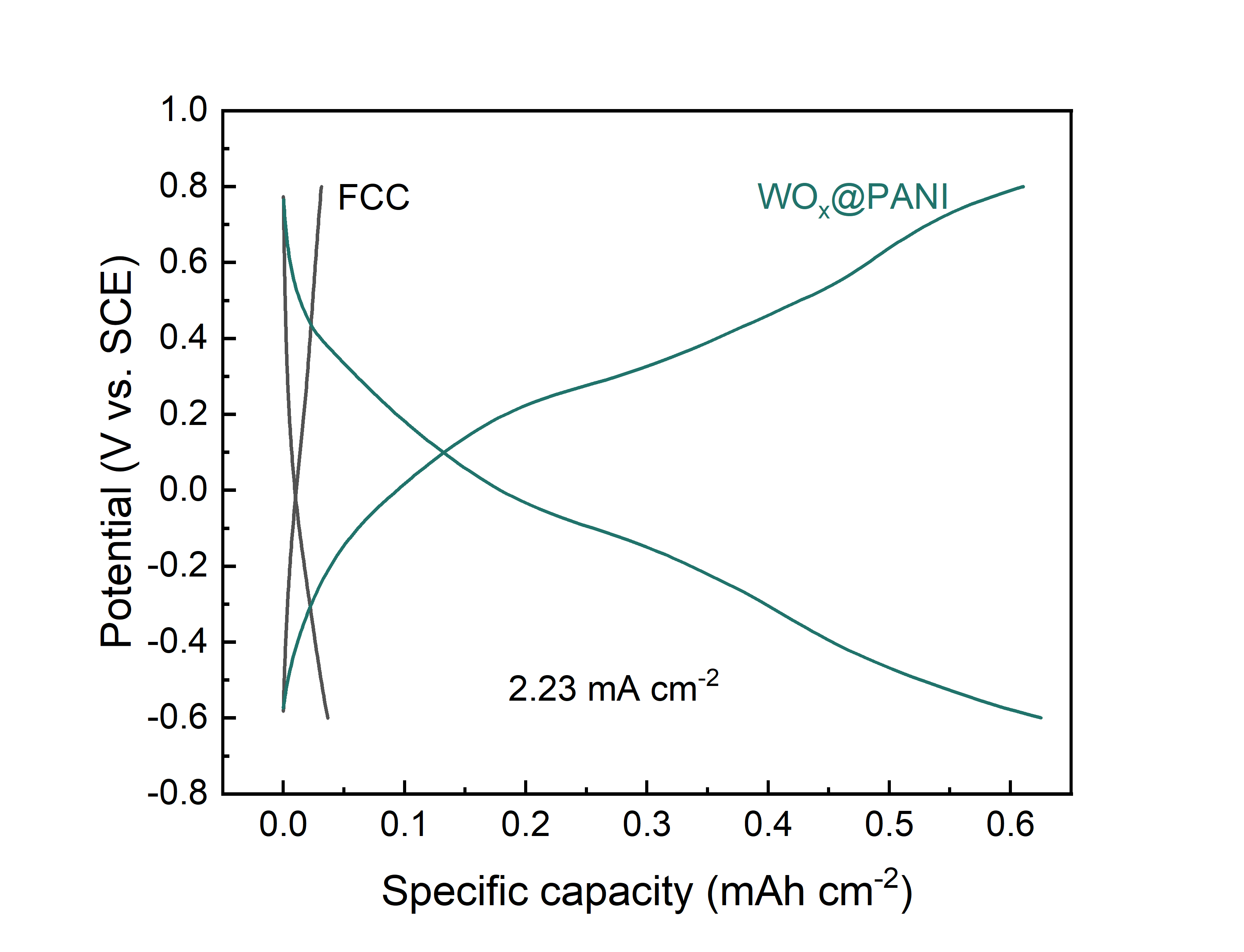


**Figure S7.** Charge/discharge curves of the FCC and WO_x_@PANI electrodes at the current density of 2.23 mA cm^-2^.


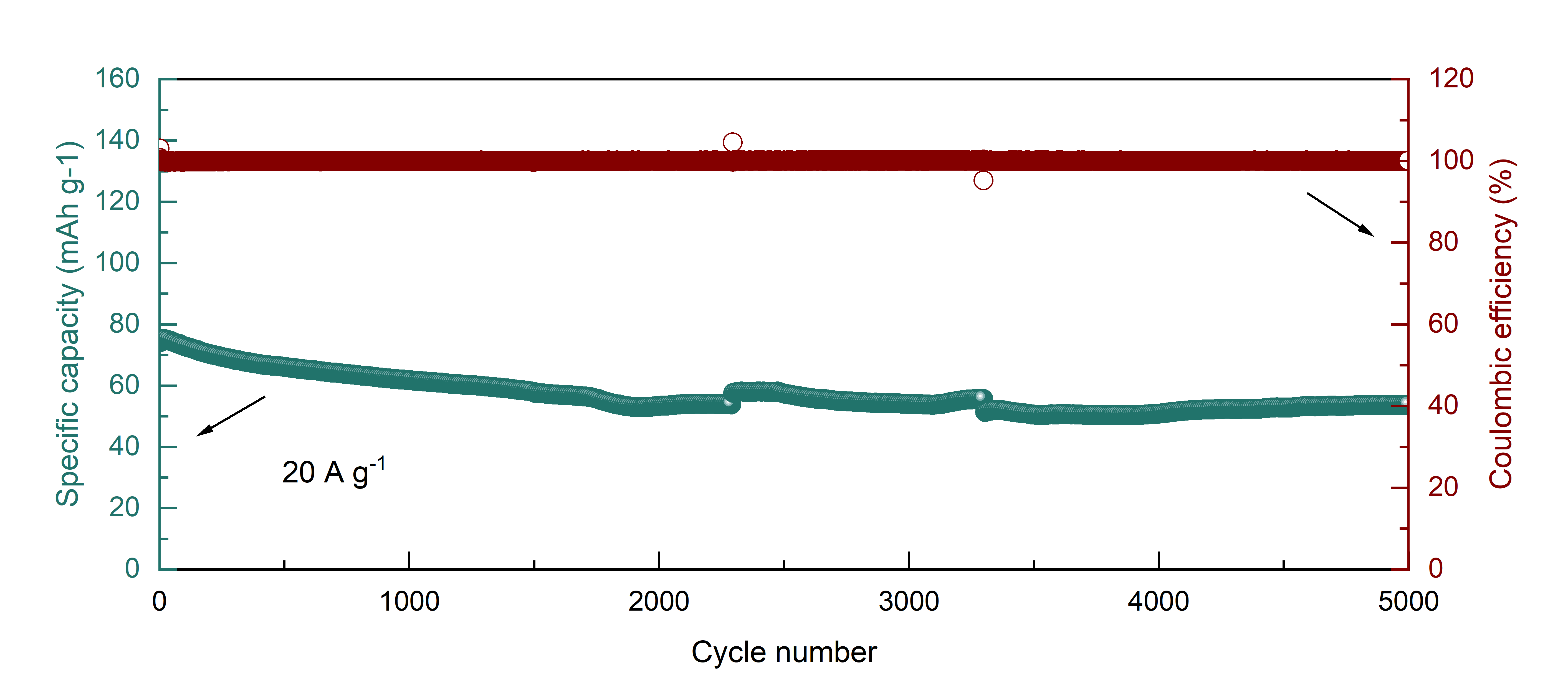


**Figure S8.** Cycle stability of WO_x_@PANI at the current density of 20 A g^−1^ after 5000 cycles.


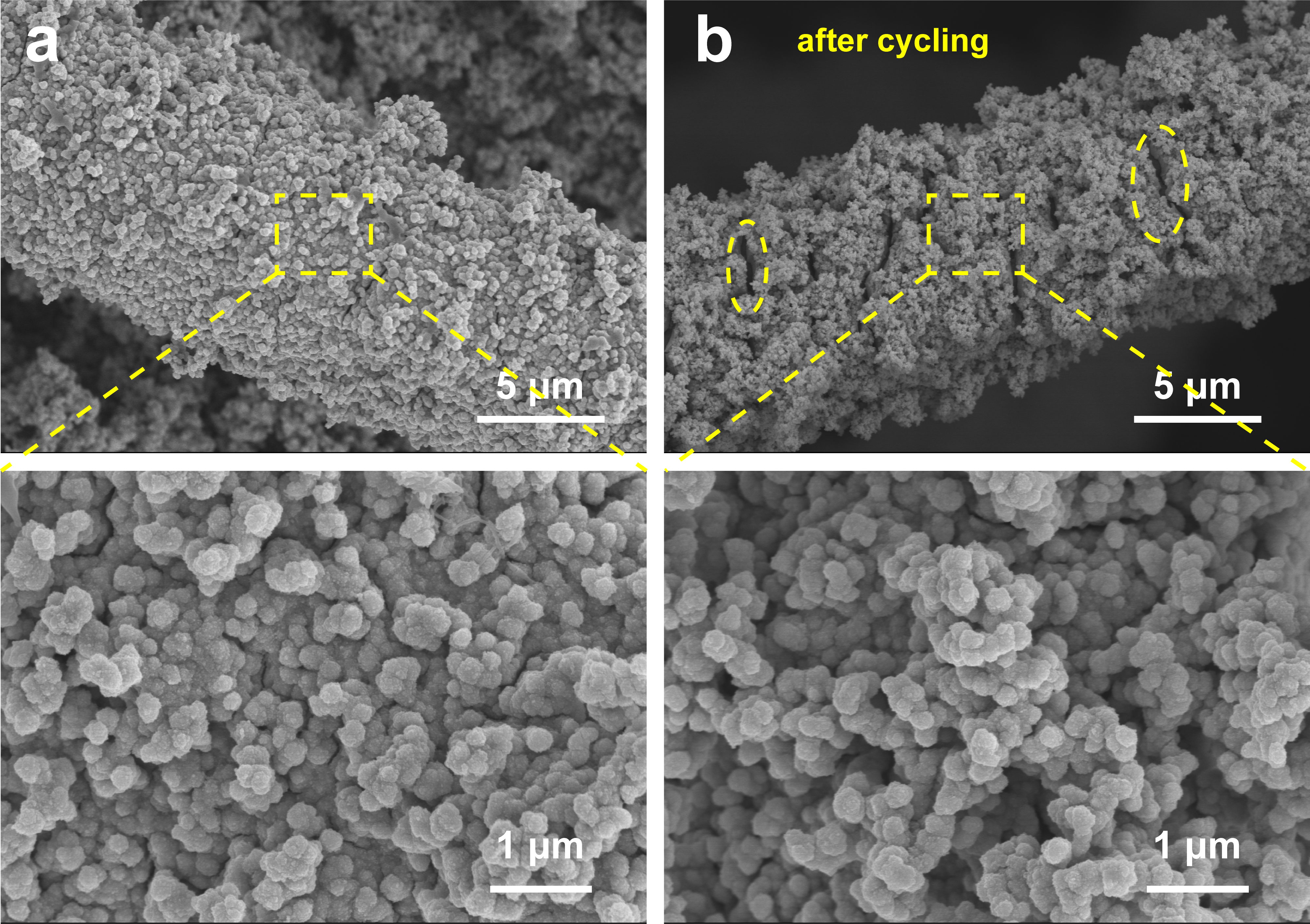


**Figure S9.** SEM images of WO_x_@PANI (a) before and (b) after high-current cycling.


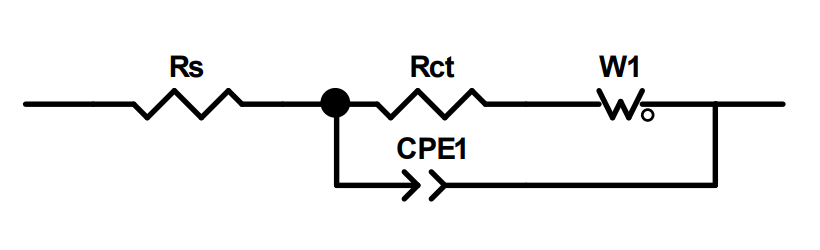


**Figure S10.** The equivalent electric circuit used to fit the Nyquist plots.

**Table S2.** The fitting results of the EIS spectra for WO_x_@PANI, WO_x_ and PANI.

| Sample | *R*_s_/Ω | *R*_ct_/Ω |
| --- | --- | --- |
| WO_x_@PANI | 1.92 | 3.81 |
| WO_x_ | 2.07 | 4.35 |
| PANI | 0.87 | 2.21 |


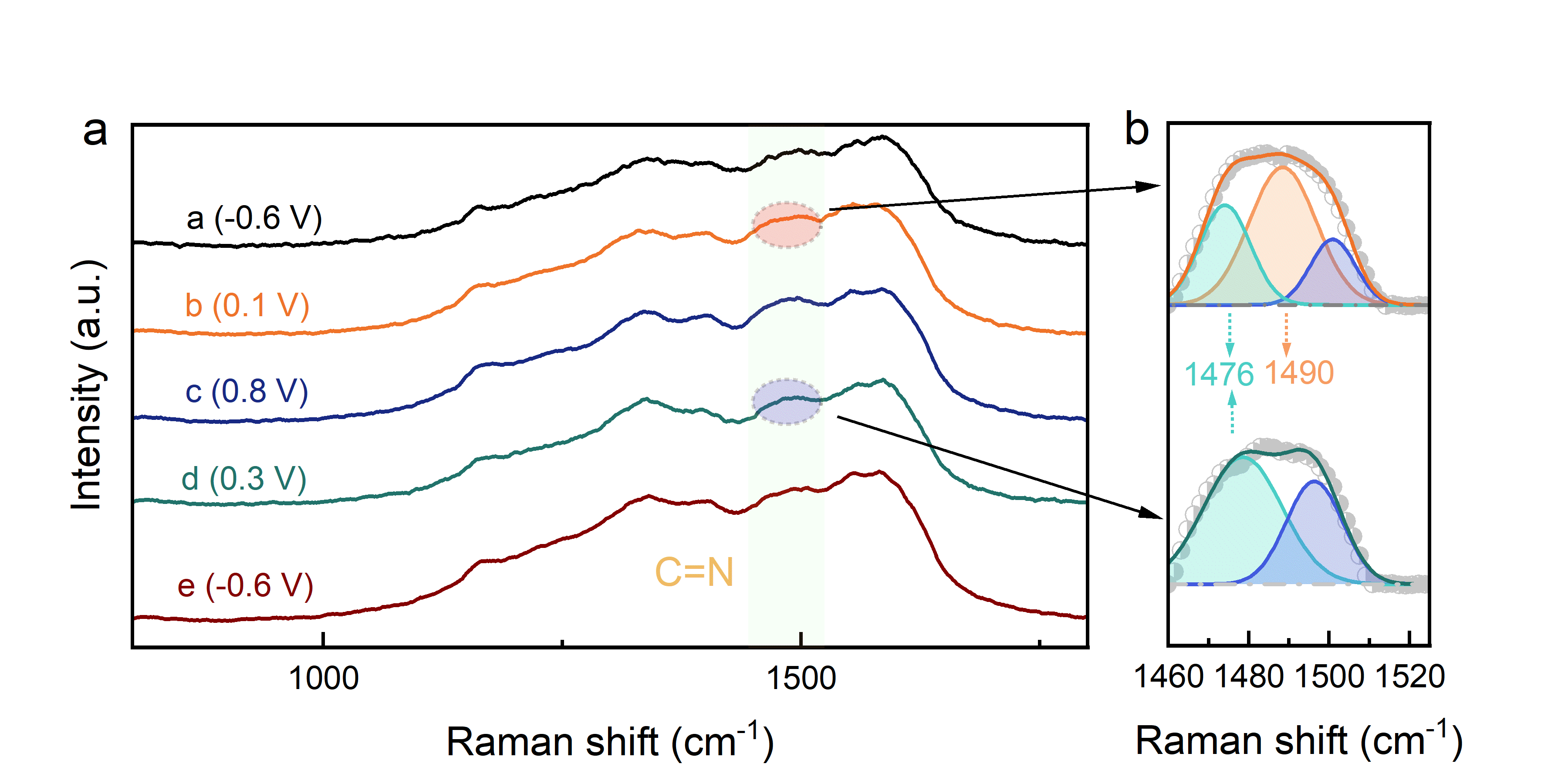


**Figure S11.** (a) Ex situ Raman spectra of PANI electrodes at different charged/discharged states. (b) Raman spectra of the charge state at 0.1 V (top) and the discharge state at 0.3 V (down).


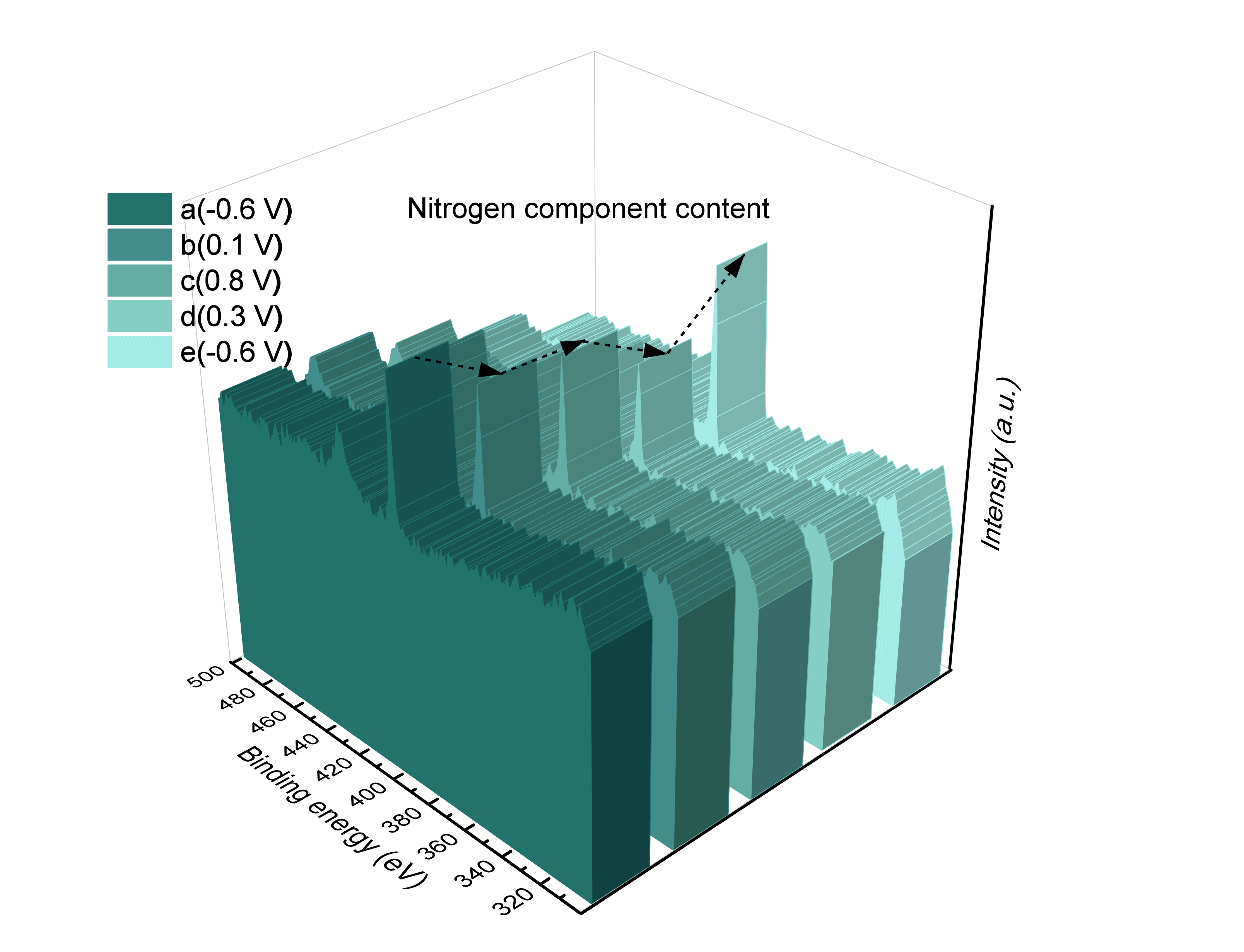


**Figure S12.** Ex situ XPS spectra of N 1s of pure PANI electrode obtained at different charging/discharging states.


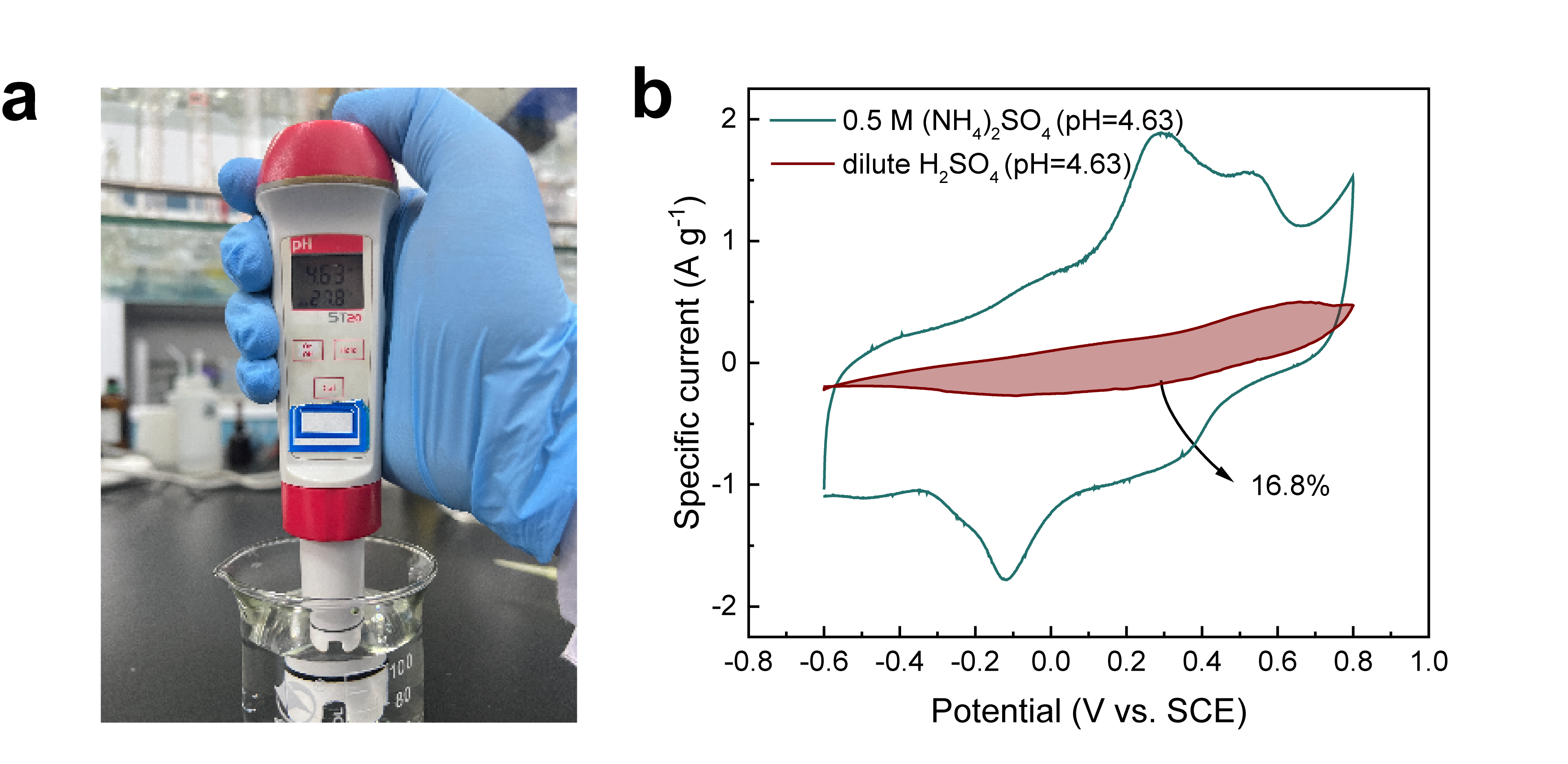


**Figure S13.** (a) Optical photograph of the pH value of dilute H_2_SO_4_. (b) CV curves of WOx@PANI in 0.5 M (NH_4_)_2_SO_4_ (pH=4.63) and dilute H_2_SO_4_ (pH=4.63) electrolyte.


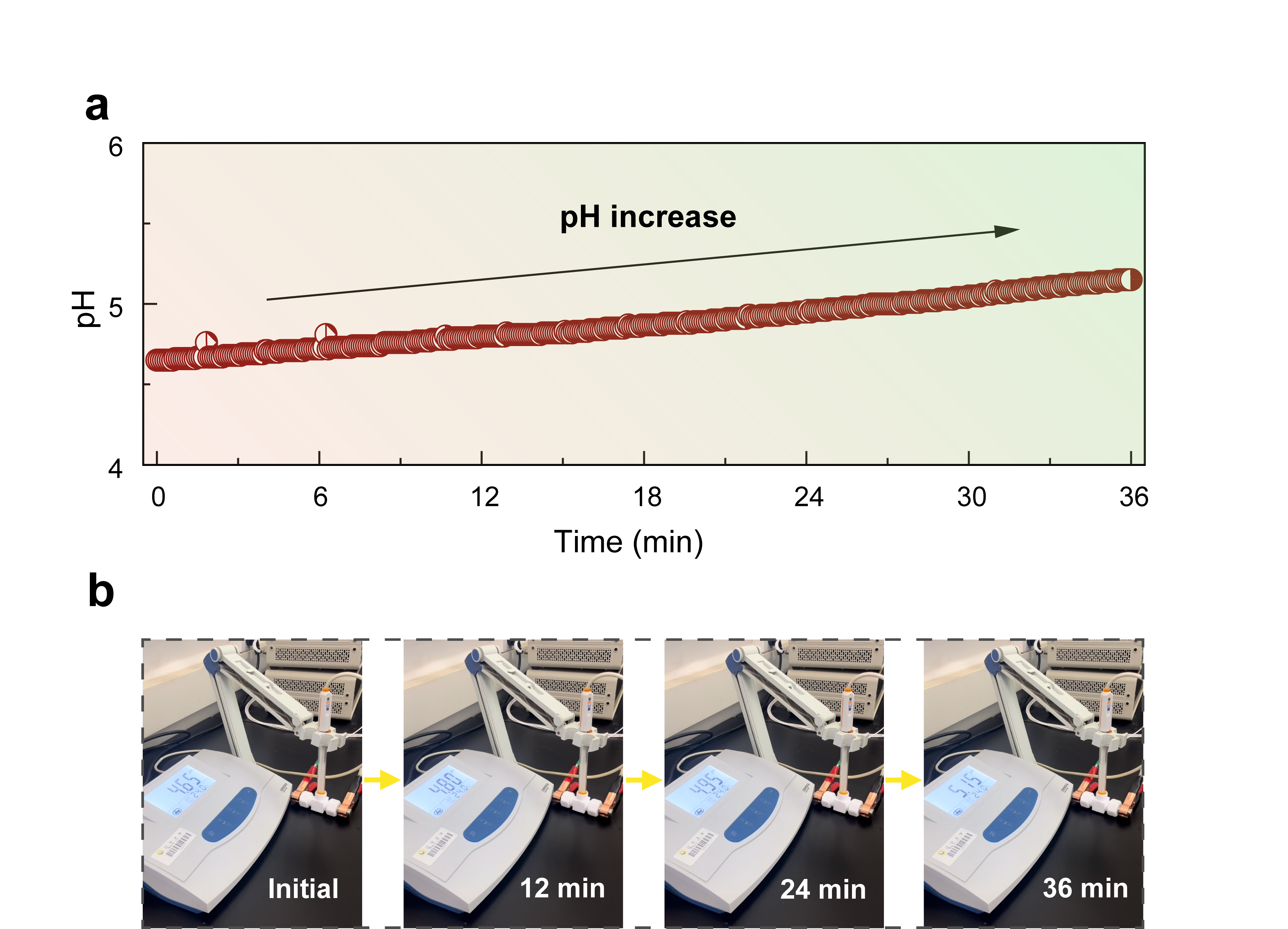


**Figure S14.** (a) pH value detection during cycling. (b) Optical photographs of pH monitoring at different times (initial, 12min, 24min and 36min).


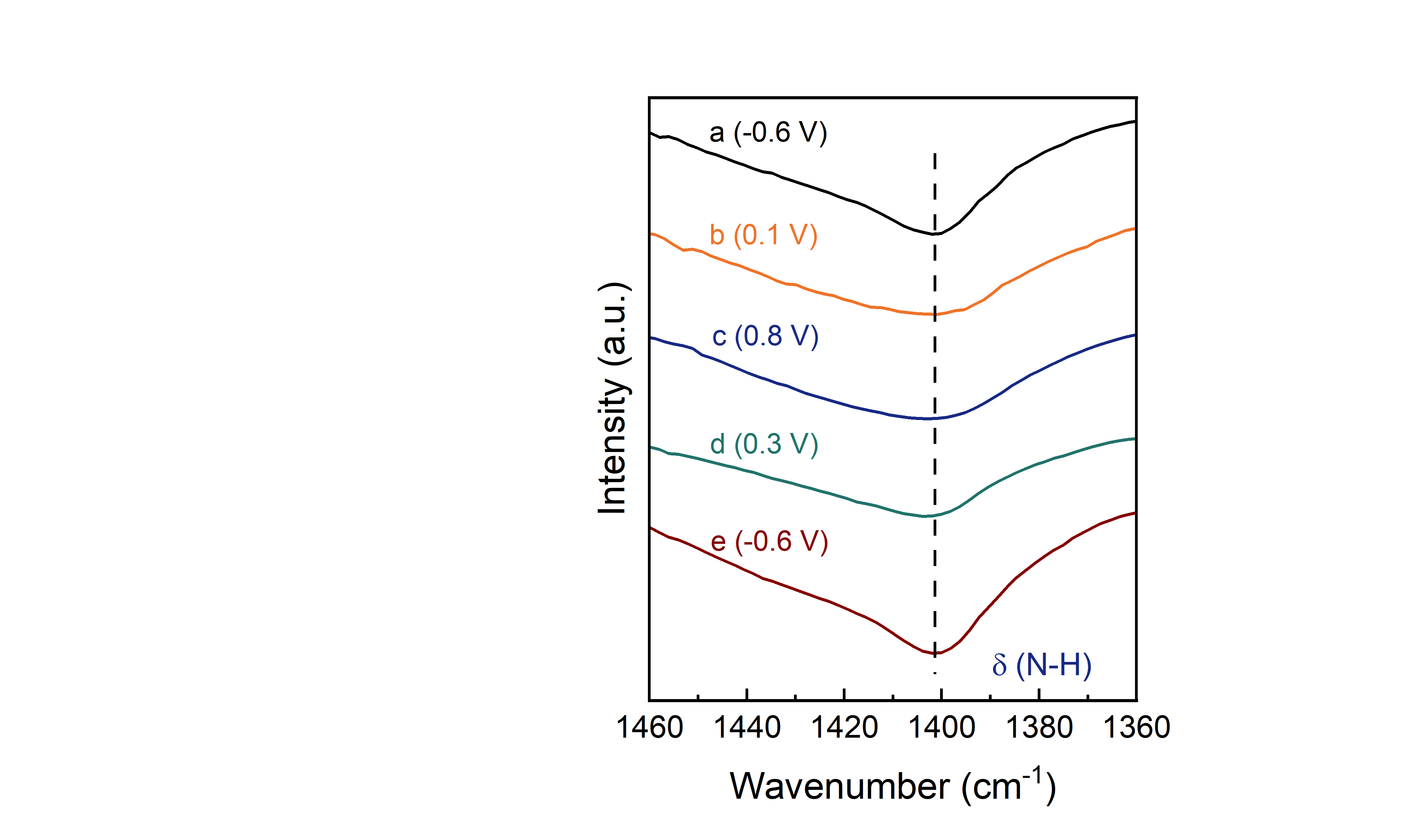


**Figure S15.** FT-IR spectra of WO_x_@PANI at various charging/discharging states. (wavenumber ranges 1360-1460 cm^-1^)


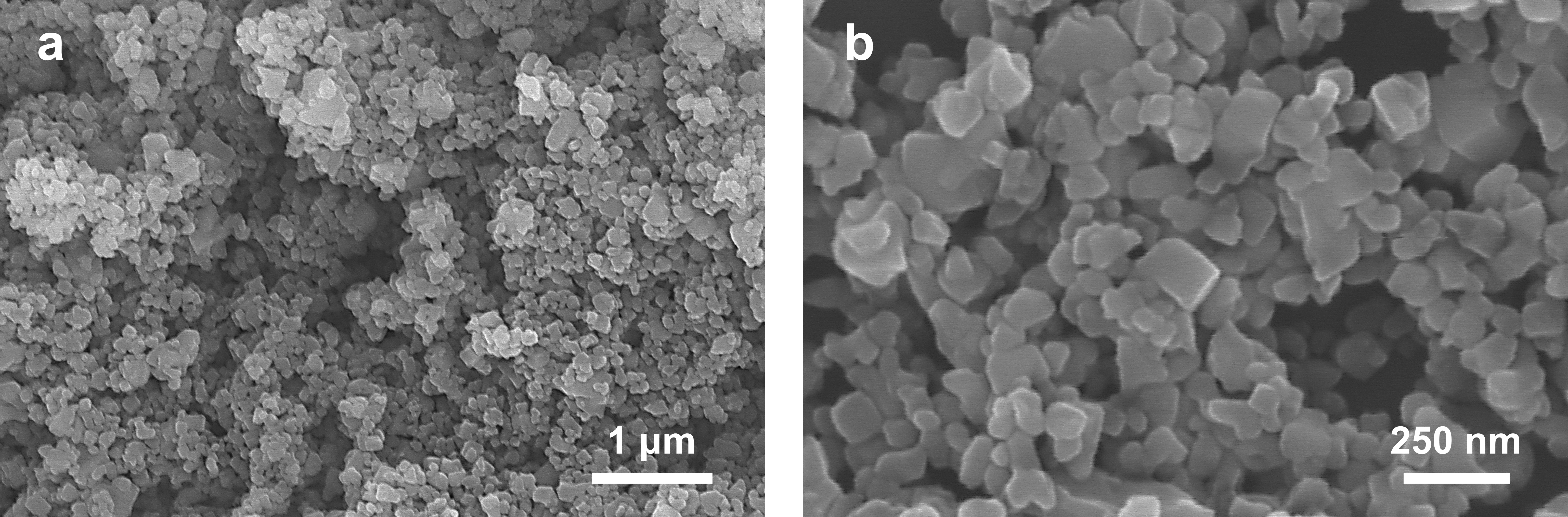


**Figure S16.** SEM images of CuFe PBA.

**
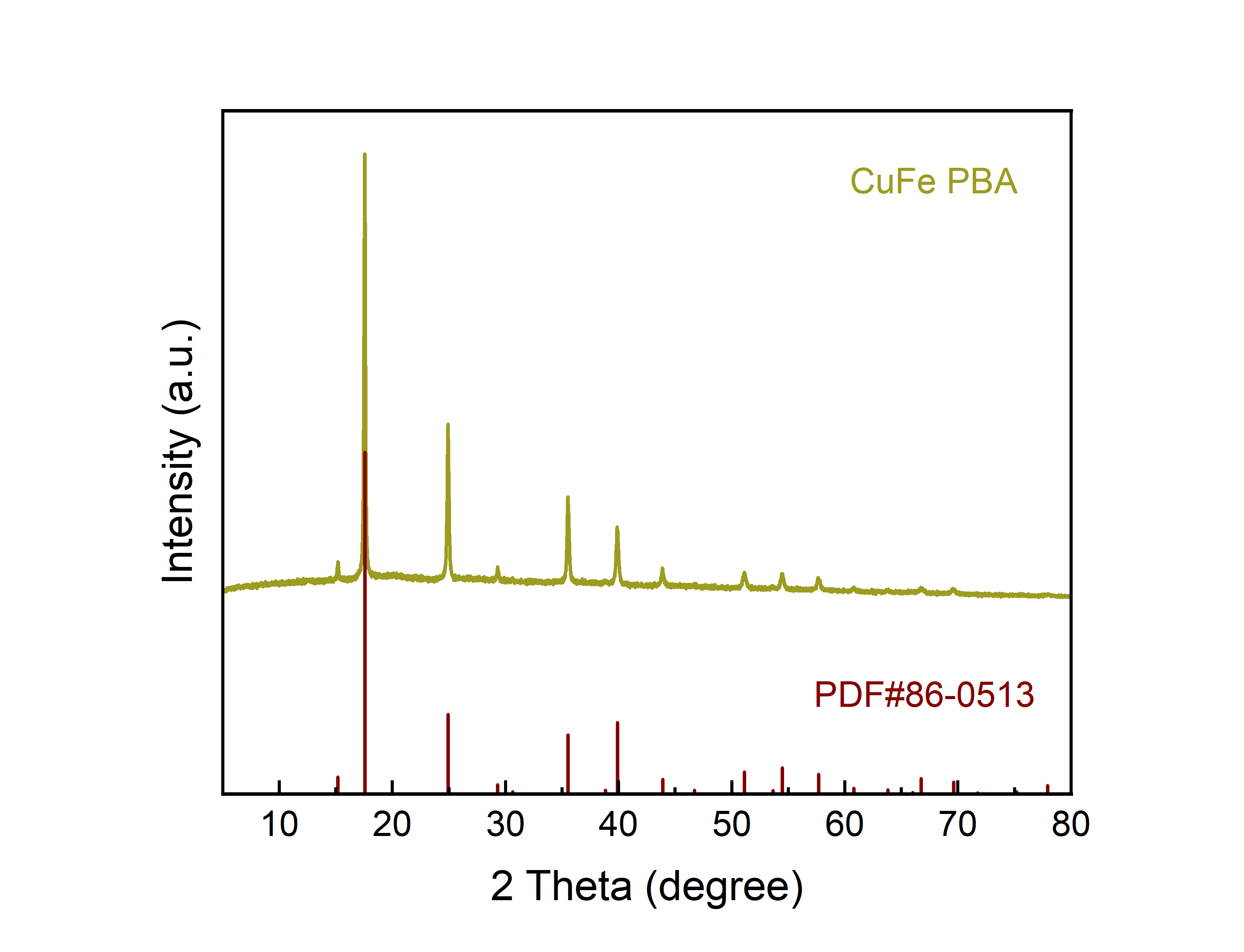
**

**Figure S17.** The XRD pattern of CuFe PBA.

**References**

[1] X. Zhang, H. Wei, B. Ren, J. Jiang, G. Qu, J. Yang, G. Chen, H. Li, C. Zhi, Z. Liu, *Adv. Mater.* **2023,** *35*, 2304209.

[2] Z. Tian, V. S. Kale, Y. Wang, S. Kandambeth, J. Czaban-Jozwiak, O. Shekhah, M. Eddaoudi, H. N. Alshareef, *J. Am. Chem. Soc.* **2021,** *143*, 19178-19186.

[3] X. Mu, Y. Song, Z. Qin, J. Meng, Z. Wang, X.-X. Liu, *Chem. Eng. J.* **2023,** *453*, 139575.

[4] G. Liang, Y. Wang, Z. Huang, F. Mo, X. Li, Q. Yang, D. Wang, H. Li, S. Chen, C. Zhi, *Adv. Mater.* **2020,** *32*, 1907802.

[5] Y. Song, Q. Pan, H. Lv, D. Yang, Z. Qin, M. Y. Zhang, X. Sun, X. X. Liu, *Angew. Chem. Int. Ed.* **2021,** *60*, 5782-5786.

[6] X. Wu, Y. Xu, H. Jiang, Z. Wei, J. J. Hong, A. S. Hernandez, F. Du, X. Ji, *ACS Appl. Energy Mater.* **2018,** *1*, 3077-3083.

[7] Y. Ma, T. Sun, Q. Nian, S. Zheng, T. Ma, Q. Wang, H. Du, Z. Tao, *Nano Res.* **2022,** *15*, 2047-2051.

[8] Y. Z. Zhang, J. Liang, Z. Huang, Q. Wang, G. Zhu, S. Dong, H. Liang, X. Dong, *Adv. Sci.* **2022,** *9*, 2105158.

[9] H. Li, J. Yang, J. Cheng, T. He, B. Wang, *Nano Energy* **2020,** *68*, 104369.
